# Microbial Functional Capacity Is Preserved Within Engineered Soil Formulations Used In Mine Site Restoration

**DOI:** 10.1038/s41598-017-00650-6

**Published:** 2017-04-03

**Authors:** Deepak Kumaresan, Adam T Cross, Benjamin Moreira-Grez, Khalil Kariman, Paul Nevill, Jason Stevens, Richard J N Allcock, Anthony G O’Donnell, Kingsley W Dixon, Andrew S Whiteley

**Affiliations:** 1 UWA School of Agriculture and Environment, University of Western Australia, 35 Stirling Highway, Crawley, WA 6009 Australia; 2 School of Plant Biology, University of Western Australia, 35 Stirling Highway, Crawley, WA 6009 Australia; 3 Kings Park and Botanic Garden, 1 Kattidj Close, Kings Park, WA 6005 Australia; 4 Department of Environment and Agriculture, Curtin University, GPO Box U1987, Bentley, WA 6102 Australia; 5 School of Pathology and Laboratory Medicine, University of Western Australia, 35 Stirling Highway, Crawley, WA 6009 Australia; 6 Faculty of Science, University of Western Australia, 35 Stirling Highway, Crawley, WA 6009 Australia; 7 Pathwest Laboratory Medicine WA, QEII Medical Centre, Monash Avenue, Nedlands, WA 6009 Australia

## Abstract

Mining of mineral resources produces substantial volumes of crushed rock based wastes that are characterised by poor physical structure and hydrology, unstable geochemistry and potentially toxic chemical conditions. Recycling of these substrates is desirable and can be achieved by blending waste with native soil to form a ‘novel substrate’ which may be used in future landscape restoration. However, these post-mining substrate based ‘soils’ are likely to contain significant abiotic constraints for both plant and microbial growth. Effective use of these novel substrates for ecosystem restoration will depend on the efficacy of stored topsoil as a potential microbial inoculum as well as the subsequent generation of key microbial soil functions originally apparent in local pristine sites. Here, using both marker gene and shotgun metagenome sequencing, we show that topsoil storage and the blending of soil and waste substrates to form planting substrates gives rise to variable bacterial and archaeal phylogenetic composition but a high degree of metabolic conservation at the community metagenome level. Our data indicates that whilst low phylogenetic conservation is apparent across substrate blends we observe high functional redundancy in relation to key soil microbial pathways, allowing the potential for functional recovery of key belowground pathways under targeted management.

## Introduction

Soil microbes are more than just indicators of ecological function, they are increasingly recognised as facilitators of the belowground metabolic recovery required for subsequent aboveground restoration^1^. Despite acknowledgement of the link between above and belowground communities^2^, there is still a lack of mechanistic understanding on how microbial communities facilitate restoration of highly degraded environments such as post-mining landscapes. For example, fungal communities are known to play a major role in stabilizing soil structure, and recent studies have demonstrated that restoration managers can exploit hyphal networks between plants and mycorrhiza^3, 4^. However, other soil functions such as C, N and P cycling arise out of a complex network of interactions between different components of highly diverse soil microbial communities and are central to aboveground plant growth and survival^5^. Little is known regarding the complexity and functions of bacterial and archaeal communities (BAC) in soils present after significant environmental degradation, such as observed in mining operations, and more critically, how they may be used in novel ways to shape the above-ground outcome of post-mining ecosystem restoration^1^.

Mine operations generate substantial volumes of waste substrates e.g. post extraction ‘tailings’, which are crushed and/or chemically treated waste rock from which ores have been extracted. Principally, they are often characterised by poor physical structure and hydrology, unstable geochemistry, and potentially toxic chemical conditions^6–8^. They represent an abundant by-product that could be used as a surrogate planting substrate for land forming, but clearly contain significant abiotic constraints to plant and microbial survival. Indeed, previous work on using nascent mine tailings alone as a habitat for microbial populations needed for aboveground restoration has focused upon the microbial community composition^9, 10^, or long-term microbial community evolution within the tailings^11, 12^. A recent microbial survey of phytostablized Pb-Zn-Cu mine tailings in a semi-arid ecosystem concluded that the imposed restoration strategy did not modify the tailings substrate sufficiently to meet the requirements of a functional soil from a microbial standpoint, and that on-going intervention in the form of nutrient amendment would be required^11, 12^.

*In situ* remediation of tailings towards a material resembling natural soils is likely to require some form of substrate amendment or accelerated weathering^11, 13^. The addition and blending of topsoil into the rhizosphere represents an effective method of alleviating some of the abiotic constraints present in pure tailings, and is likely to accelerate the return of microbial functions^7^. We hypothesized that the formation of blended ‘novel’ substrates allows a) the dilution of abiotic constraints within the tailings, b) provides a microbial inoculum from the topsoil and c) an evolving ‘soil like’ system which can be used as a planting substrate for plant ecosystem restoration. Recently Wubs and colleagues reported that application of soil inocula can promote ecosystem restoration and origins of soil inocula can play a major role in the establishment of plant communities^14^. In practice, the use of novel blended substrates for successful re-establishment of native plant communities is likely to be dependent upon the chemical characteristics of tailings and the need for dilution of these constraints at precise levels to allow a microbial ‘seed’ community from the topsoil to flourish in the new substrate. This in turn will be dependent upon the availability and characteristics of local topsoil, such as the volume available as well as the conditions of long term storage during mining, a factor which significantly affects its utility^15, 16^.

The main aim of this study was to test the efficacy of stored topsoil as a microbial inoculum to generate key soil functions relative to a local reference site harbouring established vegetation. Specifically, we assessed the phylogenetic divergence (bacterial and archaeal (BAC) communities) and functional capacity (by metagenomic approaches) within different quantitative soil blends.

## Results and Discussion

In order to assess the efficacy of blending native top soils with waste tailings to form functional planting substrates, we first established the microbial composition of the key components of the site and experimental system. This consisted of a survey of a local reference site (RS; Supplementary Fig. S1), considered the ‘pristine’ undisturbed soil state locally and the stored top soils generated during site mining operations. The reference site samples harboured a diverse range of bacterial and archaeal taxa and were dominated by sequences affiliated to the phyla *Actinobacteria, Chloroflexi, Proteobacteria* and *Crenarchaeota*. Interestingly, a large proportion of the 16S rRNA gene amplicon sequences from the RS site samples could only be assigned at the domain level (i.e. Bacteria:Other) suggesting the presence of novel or unknown lineages/taxa (Supplementary Fig S2). Similarly, the BAC composition from the stored topsoil (up to several years storage) revealed similar broad microbial compositions to the RS samples, as expected, but stored samples did reveal lower relative abundances of sequences affiliated to the phyla *Chloroflexi, Crenarchaeota* and *‘Bacteria:Other*’ in comparison to RS samples. Finally, fresh waste tailings generated from mining operations, and the material to be used for blending with stored top soils, represented a biologically inert substrate where we failed to extract any community DNA. This suggested that the tailings substrate was likely sterile and would act as a minimal source of biological input into novel ‘soil’ blended substrates.

**Figure 1:**
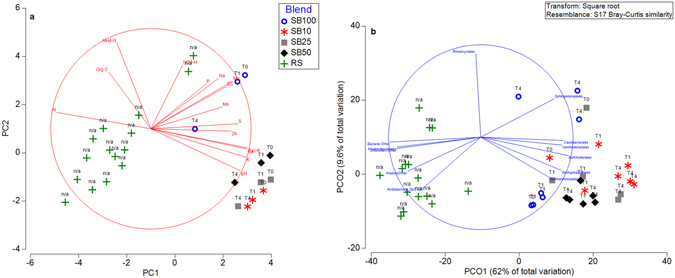
(**a**) PCA based on chemical parameters. (**b**) PCO based on bacterial and archaeal community composition (based at the level of Order). The vectors shown indicate taxa that revealed Pearson correlation values of >0.75. RS – Reference sites; SB (100, 50, 25 and 10) refers to different substrate blends. T0, T1 and T4 correspond to time zero, one week and four weeks after incubation.

The addition of alkaline tailings at a range of blends (pH > 9) resulted in significant divergence (Fig. 1a; Supplementary Tables S1) in chemistry of the substrate blends (SB) when compared to the ‘native’ reference site (RS) soil samples (pH 4). Further, when compared to stored topsoil used as the inoculum, substrate blends exhibited reduced levels of organic carbon, ammonium nitrogen and aluminium (Supplementary Table S2). For indigenous topsoil microbes, these novel substrates represented a significantly different chemical and physical environment when compared to their original habitat.

### Phylogenetic divergence in novel substrates

The proportion of topsoil used in post-mining substrate blends (i.e. the size of the initial immigrant microbial community) significantly influenced BAC composition outcomes (Fig. 1b). Significant differences were observed at the level of Order between different SB and RS samples (PERMANOVA (*P* = 0.001), ANOSIM (*R* = 0.697, *P* < 0.001). Visualisation by PCO (Fig. 1b) revealed a well-defined grouping within RS and SB samples, with stored topsoil (SB100) exhibiting a higher degree of similarity to RS samples. Although we tested different proportions of topsoil in post-mining substrates (from 10 to 50%), current leading global restoration practices (topsoil: mine waste ratios and deep ripping procedures) generally result in a rhizosphere topsoil content of at least 10% (e.g. Alcoa World Alumina Australia; www.alcoa.com/australia/en/info_page/mining_topsoil.asp). Similarity Percentage (SIMPER; Supplementary Fig. S3 and Table S3) analysis revealed that major taxa contributing to pairwise differences between SB100 and SB10 belonged to the orders *Xanthomonadales, Rhodocyclales, Sphingobacteriales, Actinomycetales and Rhodobacterales*. In addition to these taxa, sequences that can be assigned only at the domain level (i.e. ‘Bacteria:Other’) contributed significantly to the difference between RS samples and substrate blends SB100 (7.46%) and SB10 (8.35%). Amplicon sequences that were assigned only at the domain level (i.e. ‘Bacteria:Other’ and ‘Archaea:Other’) were retrieved and phylogenetically placed into a 16S rRNA gene reference tree (available through Phylosift; tree files with phylogenetic placements are available through the link https://figshare.com/s/883f61faff611052e3ab and can be visualised through the software Archaepteryx). This allowed a better resolution of the phylogenetic affiliation of unassigned sequences and clearly suggested the presence of novel taxa/lineages. For example, phylogenetic placement revealed that a major proportion of the sequences assigned only at the archaeal domain (i.e. ‘Archaea:Other’) were related to the phylum *Crenarchaetoa*. Comparison of taxonomic profiles from metagenome sequences (taxonomy assigned based on RefSeq database via MG-RAST platform; Supplementary Figs S4 and S5) revealed congruent results when compared to 16S rRNA gene amplicon sequencing, in particular with the difference in proportion of taxa between different substrate blends.

### Functional capacity in novel substrates

**Figure 2:**
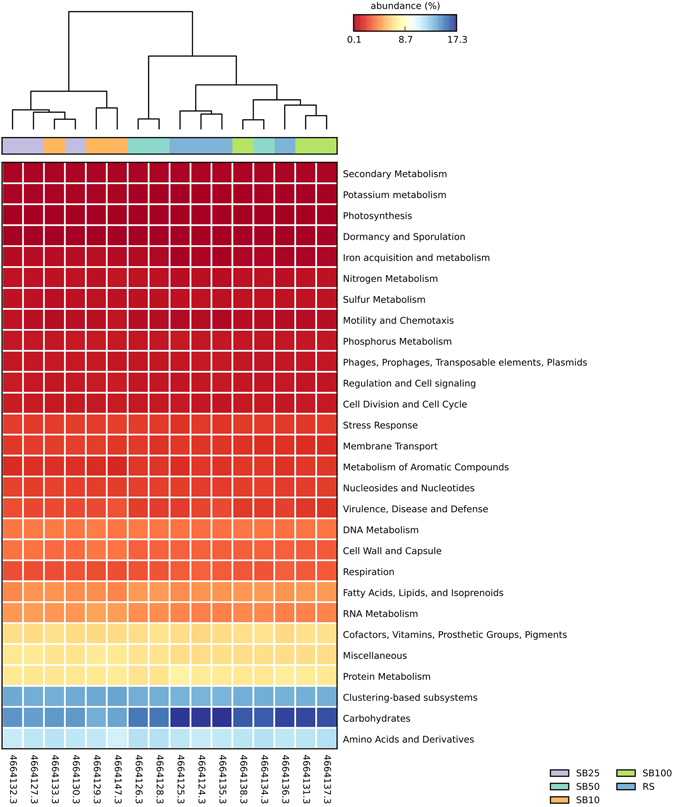
Heatmap comparing the relative abundance of metabolic genes within SEED subsystem category level 1. The columns represent the MG-RAST accession number of the metagenome and different substrate blends are colour coded.

**Figure 3:**
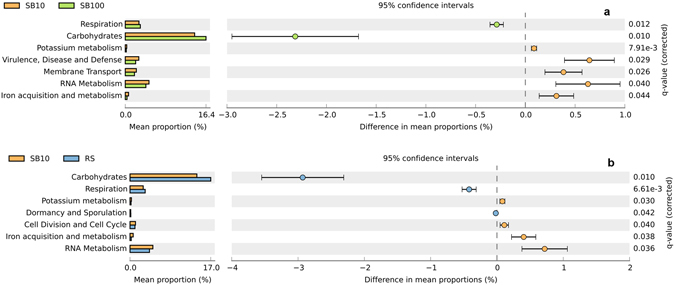
Extended bar charts representing SEED subsystem categories (at level 1) that are significantly different (P-value > 0.05) between the groups (reference site and substrate blend samples). RS represents the soil samples from the reference site and SB (10 and 100) refers to different substrate blends.

**Figure 4:**
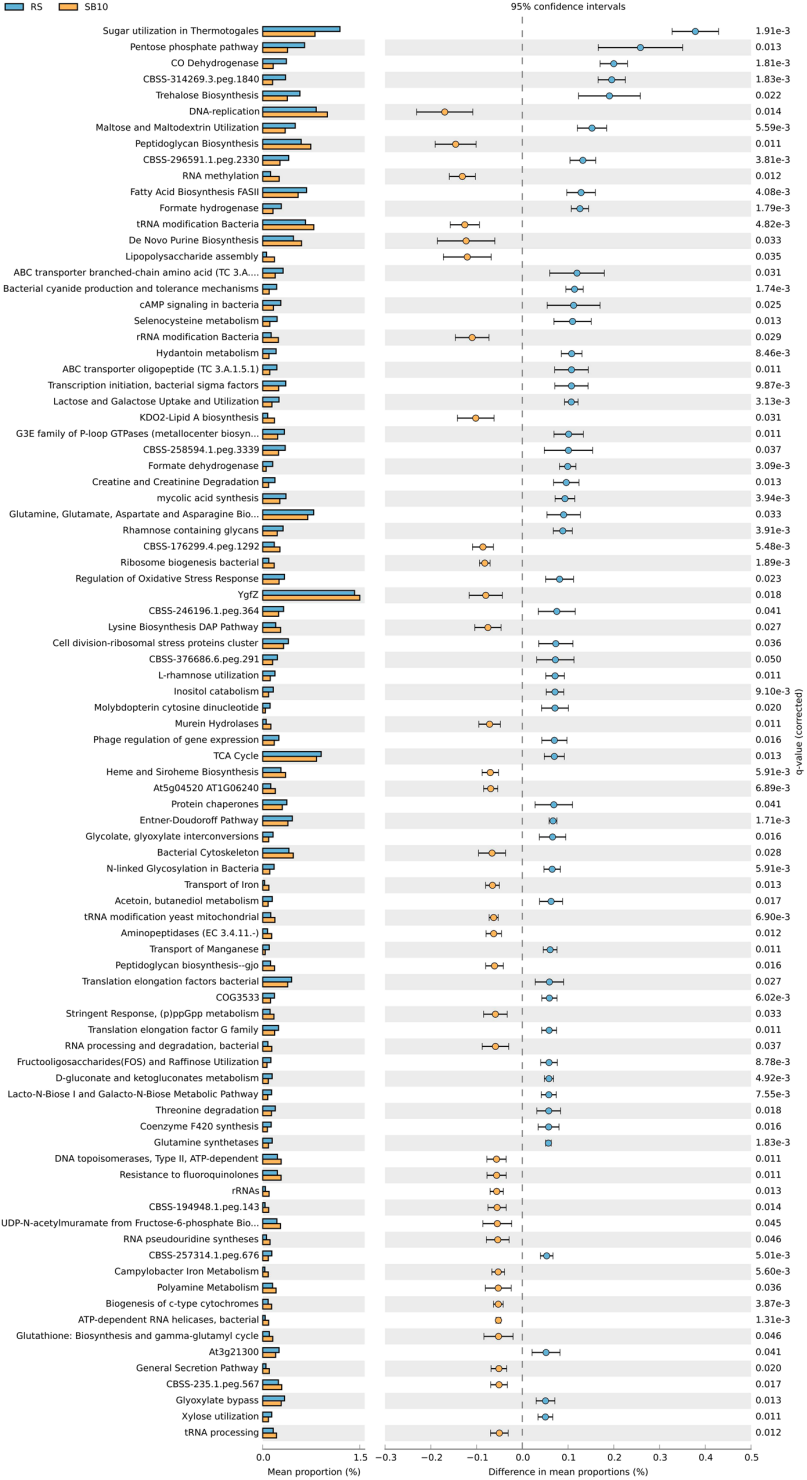
Extended bar chart representing SEED subsystem categories (at level 3) that are significantly different (P-value > 0.05 between the groups (reference site and substrate blend sample). RS represents the soil samples from the reference site and SB10 refers to different substrate blend with 10% topsoil.

**Figure 5:**
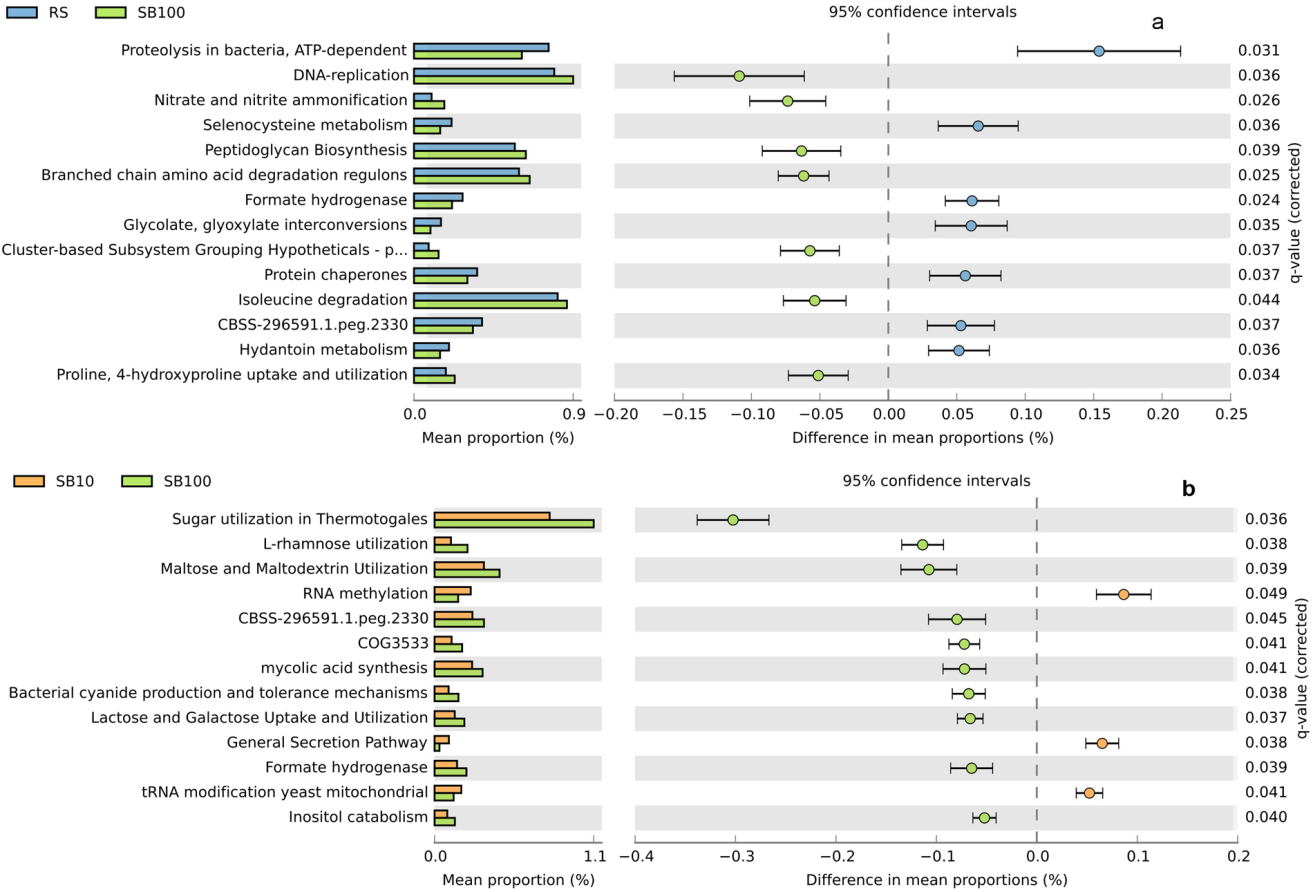
Extended bar charts representing SEED subsystem categories (at level 3) that are significantly different (P-value > 0.05 between the groups (reference site and substrate blend samples). RS represents the soil samples from the reference site and SB10 and SB100 refers to different substrate blend with 10% and 100% topsoil, respectively.

Despite differences in the BAC composition between different blending regimes, the temporal evolution of functional capacity within the blends was less stochastic. Comparison of metabolic profiles obtained from metagenomes of different SB and RS samples (Fig. 2) revealed a high degree of similarity for the relative abundance of metabolic genes (encoding for functional pathways) present across the blends, implying variable phylogenetic composition but high functional redundancy at broader levels of annotation. At SEED subsystem level 1, only marginal differences were observed in the relative abundance of metabolic genes, particularly sequences assigned to categories of carbohydrate metabolism and respiration (Fig. 3 and Supplementary Figs S6 and S7). Substrate blends SB100 and SB10 were also compared with reference site samples at a finer metabolic resolution (at SEED subsystem level 3; Fig. 4). Pairwise difference in mean proportions (>0.5% at P-value < 0.05) between SB100 versus RS and SB10 were limited in comparison to difference between RS versus SB10. For example, pairwise comparison of SB10 versus RS samples revealed that metabolic genes involved in processes such as ‘sugar utilization in Thermotogales’, ‘pentose phosphate pathway’ and ‘carbon monooxide dehydrogenase’ were over represented in RS samples among other metabolic genes (Fig. 4). Similarly, metabolic genes involved in ‘sugar utilization in Themotogales’, ‘L-rhamnose utilization’ and ‘maltose and maltodextrin utilization’ (all within Level 1 ‘Carbohydrate metabolism’) were overrepresented in SB10 in comparison to SB100 metagenome sequences (Fig. 5). From a restoration perspective, a limited difference between SB10 versus SB100 even at a finer metabolic gene resolution (at Level 3) highlighted that the functional redundancy was high even for relatively high dilutions of soil into the novel substrate and that key metabolic pathways emerge relatively quickly after blending.

### Phylogeny versus function

Soil microbial communities are known to exhibit high functional redundancy during diversity loss, both for broad functions such as decomposition or respiration^17, 18^, and for highly specialised functional guilds such as nitrifiers and denitrifiers^19^. Community level metabolic plasticity in any ecosystem relies on both phylogenetic and physiological plasticity within populations, specifically the proportion of generalists versus specialists^20^. Metabolic profiles from this study suggest a high level of functional redundancy between different SB, despite different phylogenetic compositions, possibly suggesting the prevalence of stochastic generalist populations with broad physiological capabilities. This suggests that there is an ability for the native microbial populations to adapt to extreme conditions in the post-mining substrate and potentially deliver a majority of belowground functions, given adequate time and management, which are present in the parent ‘pristine’ soil.

### Functional presence does not always mean success

It should be noted that metagenome analyses of novel substrates provide only the community metabolic potential and do not necessarily correspond to a fully functional ecosystem *per se* when compared back to the ‘pristine’ or reference site soils. However, of importance here is the observed presence of the functions under a range of novel substrate blends. If absent (or not detected) by these analyses, then clearly the function will never be expressed under any condition: the presence of a given function informs us that recovery is highly possible under the right conditions. A key factor in this recovery, and subsequent aboveground success, will be the management of this initial functional diversity to minimise future functional constraints as the soil ‘evolves’. For example, the minimisation of historical factors that reduce responses to new conditions through diversity loss^20^, such as factors which caused loss of key ecotypes/guilds which are required again as the habitat moves back to its original state. In the context of the study area this may include microbial ecotypes that are adapted to the physic-chemical factors characteristic of Western Australian soils with low pH and high Fe and Al concentration^21^, or key ecotypes/guilds which interact with native plants providing above and belowground feedback mechanisms for aboveground establishment^22^.

The use of novel substrates for successful restoration of post-mining landscapes starts with a rigorous assessment of topsoil health, the assessment of blending efficacy as well as the assessment of the evolution of key microbial ecology parameters. Here, we show that topsoil storage and the production of novel substrates influences BAC phylogenetic composition, but we also demonstrate a high level of metabolic plasticity at the community level. Specifically, we observe the presence of genes that encode for the majority of the pathways present that can be detected in the reference site soils. This clearly gives rise to the potential for functional recovery of key pathways under targeted management. This study also highlights that phylogeny-based approach should be preferred rather than only depending on taxonomic assignment of marker gene sequences if we move towards establishing ‘microbial indicators’ to assess restoration success. This strategy, together with local vegetation and soil mapping to identify key ecotypes or guilds which associate with local plant diversity will provide a sound monitoring and management basis to optimise land forming in post mining landscapes.

## Materials and Methods

### Description of the reference site

The mine site and adjacent “reference site” are located 200 km east of Geraldton in the Midwest region of Western Australia (−29.164658°, 116.786696°). Soil samples from the reference site were collected along four transects on Mt Karara Ridge (Supplementary Fig. S1), with an auger used to collect cores from the entire soil profile (0–40 cm depth). Transects were established perpendicular to the ridgeline, running southwest-northeast, with two located on each aspect of the ridge. Fifteen soil cores were sampled across transects to reflect the variation in microbial community composition in relation to aspect, elevation, and soil depth. Vegetation on the ridgeline comprised *Acacia* shrubland^23^, with dominant species including *Acacia ramulosa*, *A. assimilis, A. burkittii, Melaleuca nematophylla*, *Calycopeplus paucifolius*, and occasional *Allocasuarina acutivalvis* over mixed shrubs including *Eremophila latrobei*, *E. clarkei, Philotheca sericea, Prostanthera magnifica*, and *Aluta aspera*. All reference site soil samples for nucleic acid extraction were transported in dry ice and stored at −20 °C until further analysis.

### Experimental set-up

Substrates employed in the study comprised stored topsoil from the mining site and processed magnetite tailings (refer to Table S1 for detailed chemical characteristics), with treatments including topsoil only, tailings only, and substrate blends (SB) of topsoil:tailings at ratios of 50:50, 25:75, and 10:90. Soil ratio mixes were homogenized prior to experimental use. Replicate 40 mm plastic tubestock pots were filled with each substrate, with five replicates sampled for each SB at each sampling time. Pots were incubated at constant 30 °C under a 12-hour light/dark cycle for four weeks, with temperature regime selected to be reflective of average local climatic conditions during March–April (http://www.bom.gov.au/climate/averages/tables/cw_008093.shtml). Landforming and topsoil return are generally undertaken during this period each year, prior to seed broadcast and restoration works. All pots were watered daily with 20 ml of deionised water (pH 6.0 ± 0.1) to maintain soils at field capacity (ca. 23% gravimetric moisture content). Soil samples were taken prior to incubation (T0), and destructively sampled at one-week intervals for four weeks (samples from T0, T1 – one week and T4 – four weeks were subsequently used for downstream processing), with the top 2 cm of substrate from each replicate transferred to 15 ml tubes and all remaining substrate from each treatment pooled into a single sample for chemical analysis. All soil samples were stored at −20 °C until analysis.

### Chemical analysis of substrate blends

To examine the chemical composition of RS and SB (Table S1), 400 g samples of all SB were sent to CSBP Plant and Soil Laboratories (Bibra Lake, Western Australia) for analytical determination of chemical factors including ammonium nitrogen (NH_4_-N), nitrate nitrogen (NO_3_-N), phosphorus colwell (P), potassium colwell (K), sulfur (S), organic carbon (Org C), salinity (EC), pH-CaCl_2_, copper (Cu), iron (Fe), manganese (Mn), zinc (Zn), aluminium (Al), calcium (Ca), magnesium (mg) potassium-exc (K-exc), sodium (Na) and boron (B).

### DNA extraction, PCR amplification and bioinformatic analysis

DNA was extracted from 0.25 g of soil samples from both RS and SB using a Powersoil-htp^96^ Soil DNA isolation kit (MO BIO laboratories, CA) following the manufacturer’s instructions. Repeated attempts to extract DNA from pure tailings failed and were subsequently excluded from further analysis. DNA was quantified using a QUBIT 2.0 fluorometer (Life Technologies, USA) and subsequently used as the template for PCR amplification of the 16S rRNA gene (V4 region) using the universal PCR primer set 515F and 806R targeting members within both bacterial and archaeal domain. The forward primer included the addition of an Ion Torrent PGM sequencing adapter, a GT space and unique Golay barcode to facilitate multiplexed sequencing. The barcoded PCR reaction (20 μl) consisted of; template DNA (1 ng), universal primer mix (untagged 515 F and 806 R at a final concentration of 0.2 μM), tagged 515 F primer (0.2 μM), 600 ng BSA (Life Technologies) and 2.5 × 5Primer Hot Master Mix (5 Primer, Australia). The PCR reactions conditions were 94 °C for 2 min (denaturation), followed by 25 cycles of denaturation at 94 °C for 45 seconds, annealing at 53 °C for 60 seconds, elongation at 72 °C for 90 seconds and a final extension step at 72 °C for 10 minutes. Following PCR amplification, PCR products were checked for size and specificity by electrophoresis on a 2.5% w/v agarose gel, purified using Ampure (Beckman Coulter, Australia), quantified and pooled for multiplex sequencing on an Ion Torrent PGM. Quality filtered sequences were subsequently analysed through the Quantitative Insights into Microbial Ecology (QIIME) pipeline^24^. Briefly, the quality parameters used for analysis were; minimum average quality score of 20, minimum sequence length of 130 bp, maximum sequence length of 350 bp, no primer mismatch or barcode error allowed, maximum length of homopolymers was 15 and maximum number of ambiguous bases was six. De novo OTU picking was performed using uclust at 97% sequence identity and subsequently taxonomy was assigned to each OTU based on the Greengenes database (version 13.8). The resulting OTU tables at different levels were used as measures of taxa relative abundance in multivariate statistical analysis (MVS) analysis. A total of 563976 reads were obtained after QC and removal of chimeric sequences using Usearch v6.1, which were grouped into 22245 OTUs after removal of singletons and doubletons. Phylosift^25^ was used to infer phylogeny of sequences that were assigned only at the domain level in the QIIME pipeline. Both bacterial and archaeal trees are available in phyloXML format through https://figshare.com/s/883f61faff611052e3ab that can be visualised interactively through the software Archaeopteryx.

Shotgun metagenome sequencing was performed using the Ion Proton platform^26^ using pooled DNA samples from replicates, representing four substrate blends from three time points 0, 1 and 4 (i.e. 12 samples) alongside four soil samples from the reference site. The metagenome sequences from the sixteen samples were uploaded to the Metagenome Rapid Annotation using subsystem Technology (MG-RAST; http://metagenomics.anl.gov) server and annotated using the SEED database for functional classification^27, 28^.

### Statistical analysis

The effect of different substrate blends and time on BAC composition was visualised through principal coordinates analysis (PCO) using the Bray-Curtis measure of similarity and for environmental parameters using the principal component analysis (PCA). The PERMANOVA model (using default settings) used a two-way factorial design using factors “blend” and “time” and their interaction to test for significance. Similarity percentage (SIMPER) routine was used to identify taxa (at the level of Order) that explained significant differences in BAC composition between different substrate blends and reference site samples (Table S3). All tests were conducted at α = 0.05. Heat map plots of BAC composition (Fig. S2) were generated using the pheatmap package v1.0.8 in R (R Development Core Team, 2011). The OTU output table from QIIME was exported and filtered in R, according to the abundance of each taxon, i.e. where taxa with less than 25% of the whole matrix mean were rejected (<1.08% and <0.22% for Phylum and Order level, respectively; SF 2A and 2B). Multivariate statistical analyses were performed using Primer-E 7 software with PERMANOVA + packages^29^. Comparison of statistical differences between metabolic and taxonomic profiles from metagenome sequences was performed using STAMP – statistical analysis of metabolic profiles^30^ with profiles imported from MG-RAST (maximum e-value cutoff was 1e-5, minimum identity cutoff was 60% and minimum alignment length was 15; Subsystems database for metabolic profiles and RefSeq database for taxonomic profiles). Welch’s t-test was used to compare the proportions of metabolic genes between two groups using the method of Welch’s inverted with the correction of Benjamini-Hochberg for false discovery rate^31, 32^. The soil chemical data were subjected to one-way ANOVA using the Statistical Analysis System (SAS) version 9.2 (SAS Institute, Inc., Cary NC, USA) software package and means were separated using LSD at 5% significance level.

## Electronic supplementary material

Supplementary information
