# Supplementary material for: Microbial Functional Capacity Is Preserved Within Engineered Soil Formulations Used In Mine Site Restoration
Source: Sci Rep. 2017 Apr 3;7:564. doi: 10.1038/s41598-017-00650-6 (PMC5428872; doi:10.1038/s41598-017-00650-6)
Supplement: Supplementary file 1 — Supplementary information [file 41598_2017_650_MOESM1_ESM.pdf]

Supplementary information

Microbial Functional Capacity Is Preserved Within Engineered Soil Formulations Used In Mine Site Restoration

Kumaresan D, Cross AC, Moreira-Grez B, Kariman K, Nevill P, Steven J, Allcock R, O'Donnell AG, Dixon KW and Whiteley AS

Supplementary figure legends

Figure S1: Location of mine site and the adjacent reference site. The sampling locations for reference site samples are indicated with pins (different colours represent different transects).

Figure S2: Heat map comparing relative abundance of taxa (at the Order level) between different substrate blends and reference site samples. RS– Reference site samples; SB (100,50, 25 and 10) refers to different substrate blends. T0, T1 and T4 correspond to time zero, one week and four weeks after incubation.

Figure S3: Schematic representation of the dissimilarity percentages between reference site (RS) samples and different substrate blends (SB) based on similarity percentage (SIMPER) analysis of BAC composition (based at the level of Order). The width of the ribbon connecting two groups represents the percentage of dissimilarity.

Figure S4: Stacked bar chart representing the difference in taxa within all domains (at the phylum level) based on metagenome sequences (annotated using RefSeq database).

Figure S5 (a-j): Extended bar charts representing difference in proportion of taxa based on metagenome sequences (at order level and greater than 0.5%) that are significantly different (P-value > 0.05) between the groups (reference site and substrate blend samples). RS represents the soil samples from the reference site and SB (10, 25, 50 and 100) refers to different substrate blends.

Figure S6: Phylogenetic placement of 16S rRNA gene sequences that were assigned to the archaeal domain. Diameter of the blue circles represent the relative abundance of sequences placed in that particular position.

Figure S7: Principal co-ordinates plot based on metabolic gene profiles obtained from different metagenomes (at SEED subsystem Level 3). RS represents the soil samples from the reference site and SB (100. 50, 25 and 10) indicates different substrate blends.

Figure S8: Extended bar charts representing SEED subsystem categories (at level 1) that are significantly different (P-value > 0.05) between the groups (reference site and substrate blend samples). RS represents the soil samples from the reference site and SB (25, 50 and 100) refers to different substrate blends.

Fig S1

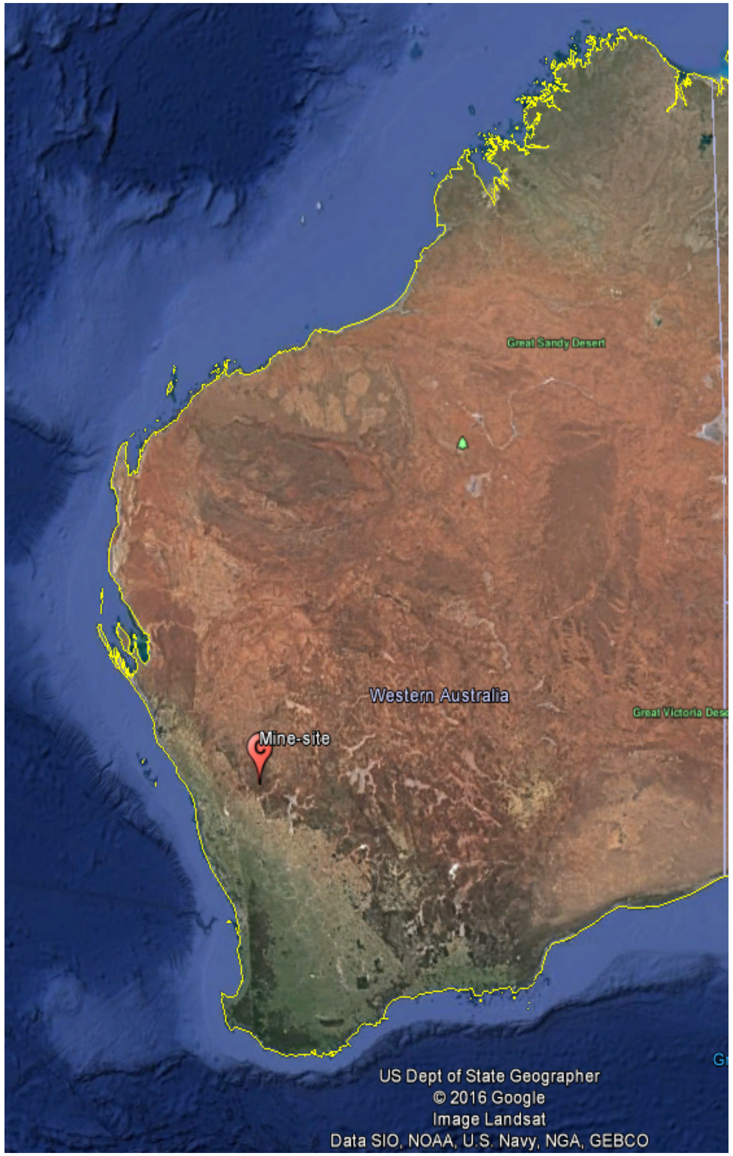
Great Sandy Desert
Western Australia
Great Victoria Dese
Mine-site
US Dept of State Geographer
© 2016 Google
Image Landsat
Data SIO, NOAA, U.S. Navy, NGA, GEBCO

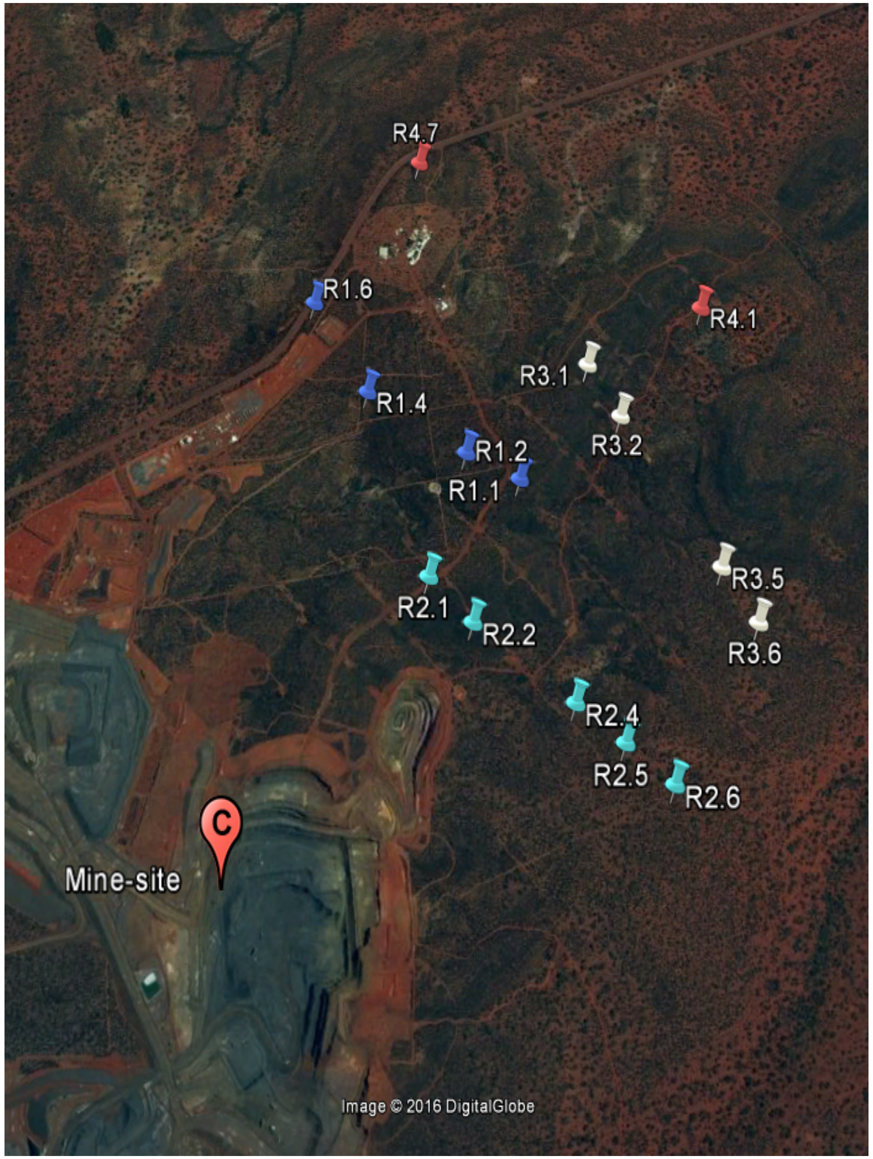
R4.7
R1.6
R4.1
R3.1
R1.4
R3.2
R1.2
R1.1
R3.5
R2.1
R2.2
R3.6
R2.4
R2.5
R2.6
C
Mine-site
Image © 2016 DigitalGlobe

Fig S2

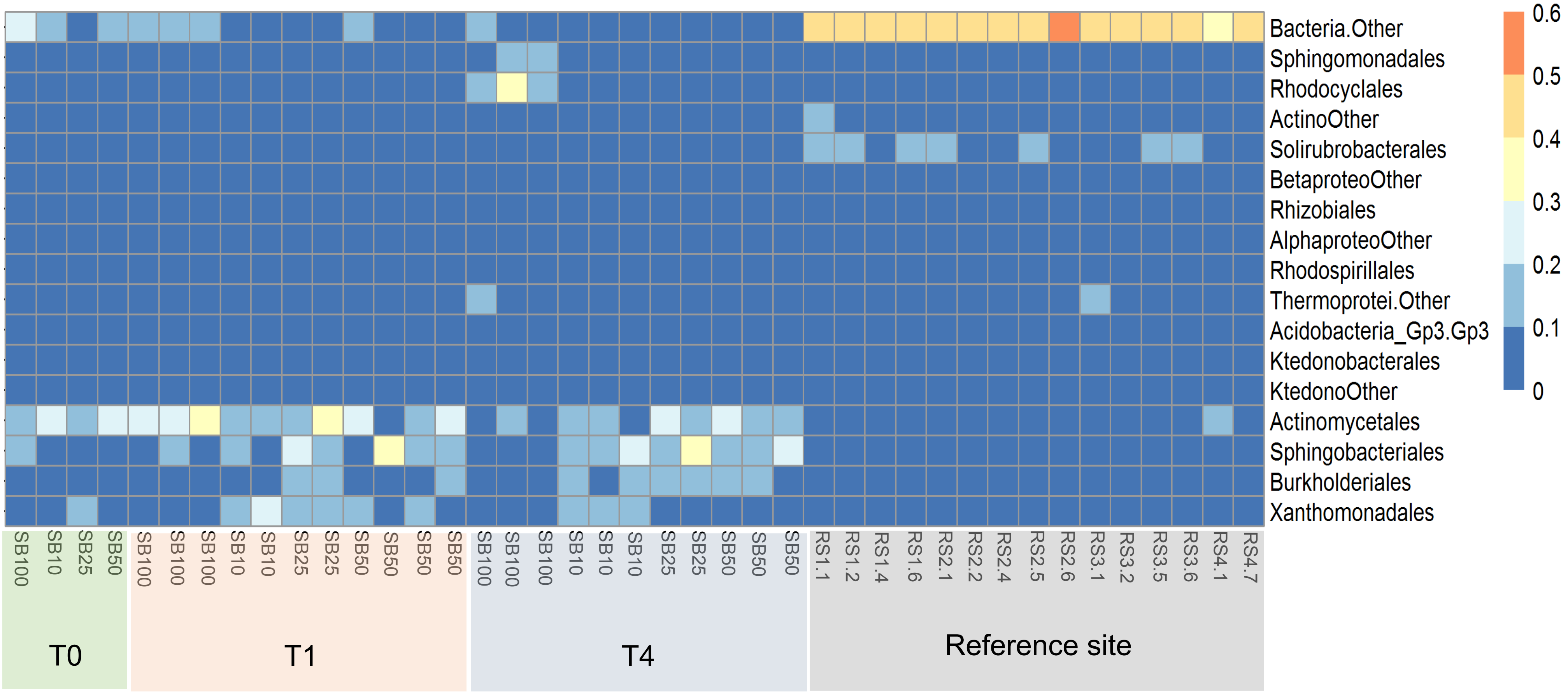

Bacteria.Other
Sphingomonadales
Rhodocyclales
ActinoOther
Solirubrobacterales
BetaproteoOther
Rhizobiales
AlphaproteoOther
Rhodospirillales
Thermoprotei.Other
Acidobacteria_Gp3.Gp3
Ktedonobacterales
KtedonoOther
Actinomycetales
Sphingobacteriales
Burkholderiales
Xanthomonadales
0.6
0.5
0.4
0.3
0.2
0.1
0
SB100
SB10
SB25
SB50
SB100
SB100
SB100
SB10
SB10
SB25
SB25
SB50
SB50
SB50
SB50
SB100
SB100
SB100
SB10
SB10
SB10
SB25
SB25
SB50
SB50
SB50
RS1.1
RS1.2
RS1.4
RS1.6
RS2.1
RS2.2
RS2.4
RS2.5
RS2.6
RS3.1
RS3.2
RS3.5
RS3.6
RS4.1
RS4.7
T0
T1
T4
Reference site

Fig S3

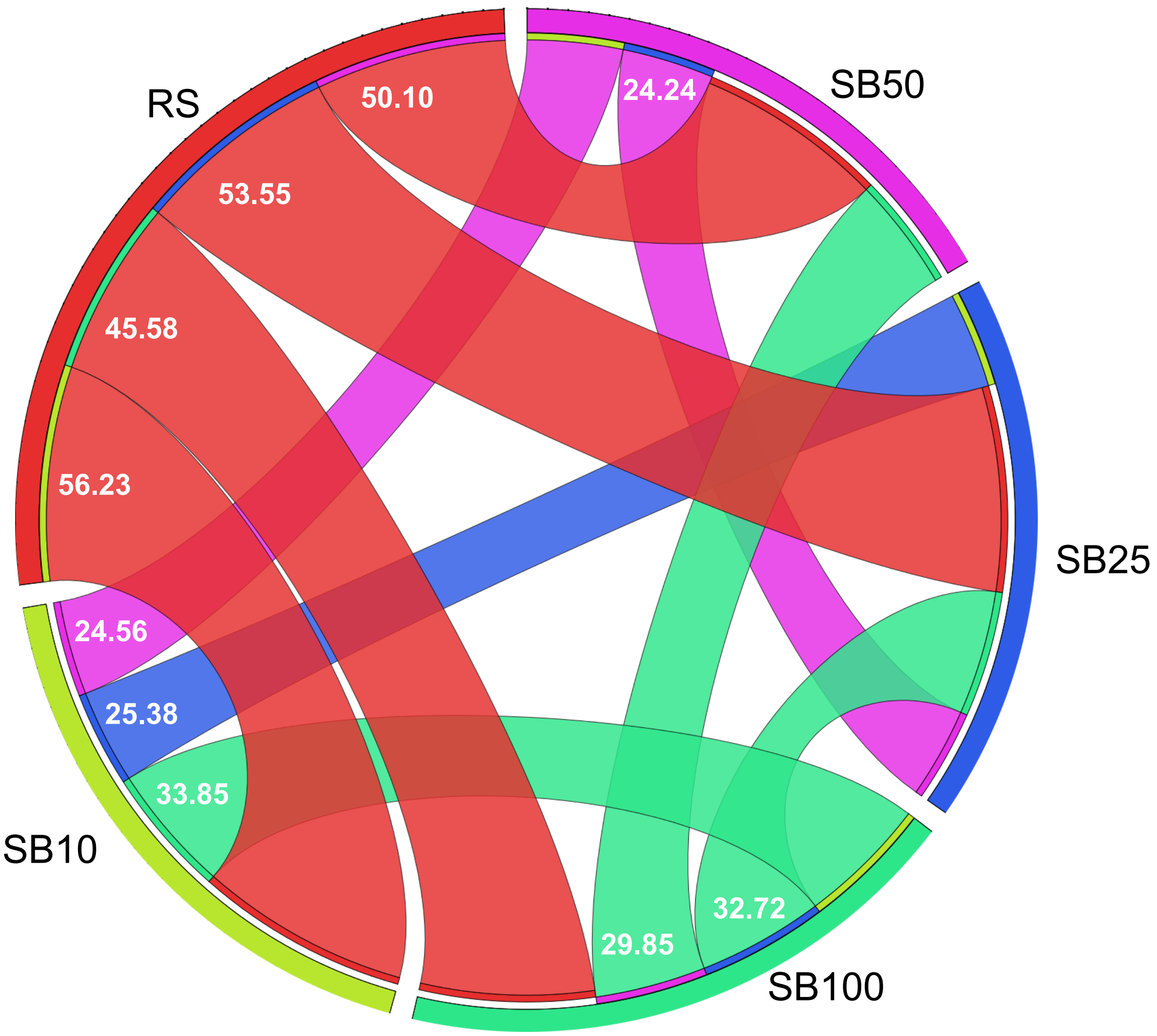

RS
SB50
SB25
SB100
SB10
50.10
24.24
53.55
45.58
56.23
24.56
25.38
33.85
32.72
29.85

Fig S4

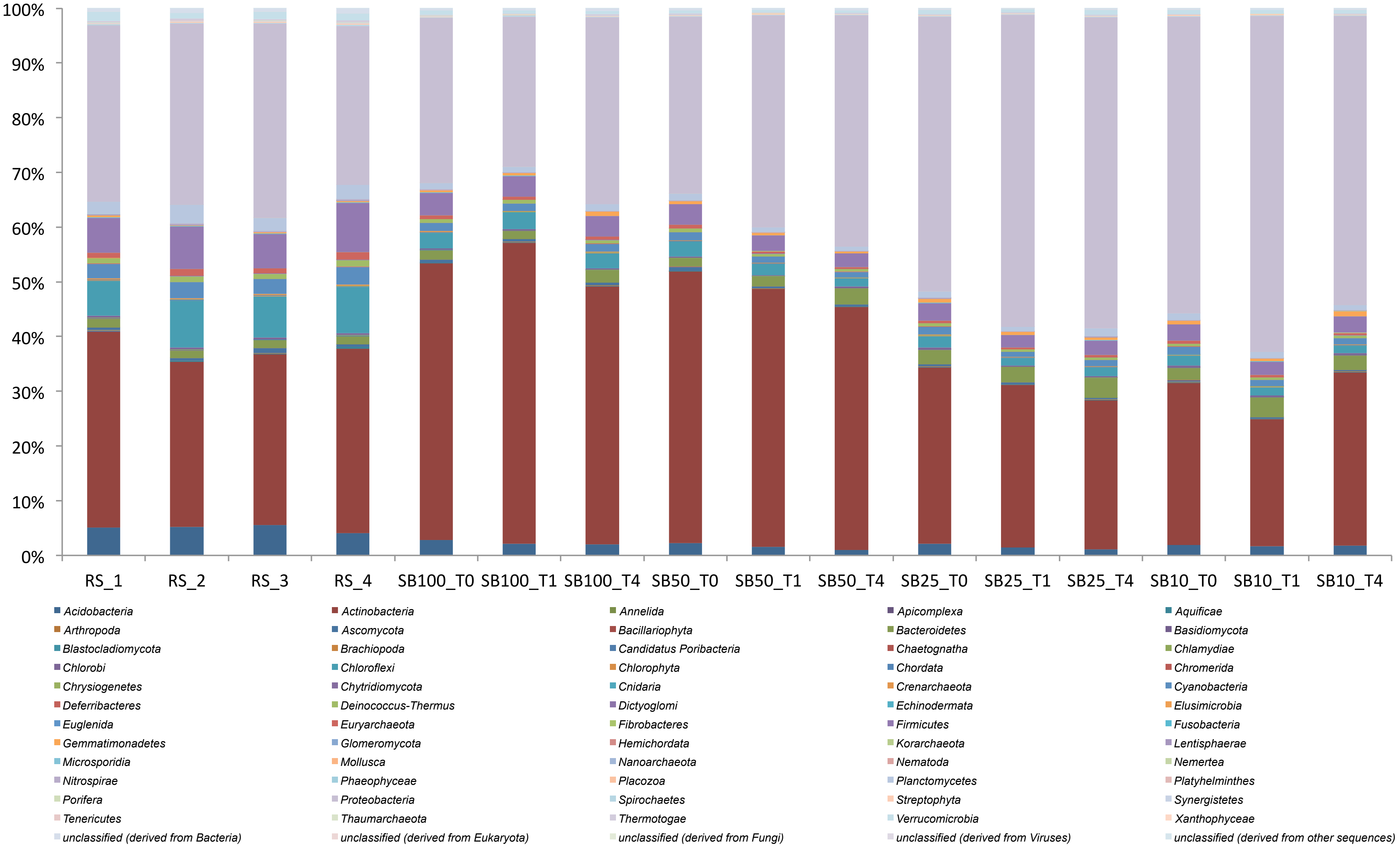
100%
90%
80%
70%
60%
50%
40%
30%
20%
10%
0%
RS_1
RS_2
RS_3
RS_4
SB100_T0
SB100_T1
SB100_T4
SB50_T0
SB50_T1
SB50_T4
SB25_T0
SB25_T1
SB25_T4
SB10_T0
SB10_T1
SB10_T4
Acidobacteria
Actinobacteria
Annelida
Apicomplexa
Aquificae
Arthropoda
Ascomycota
Bacillariophyta
Bacteroidetes
Basidiomycota
Blastocladiomycota
Brachiopoda
Candidatus Poribacteria
Chaetognatha
Chlamydiae
Chlorobi
Chloroflexi
Chlorophyta
Chordata
Chromerida
Chrysiogenetes
Chytridiomycota
Cnidaria
Crenarchaeota
Cyanobacteria
Deferribacteres
Deinococcus-Thermus
Dictyoglomi
Echinodermata
Elusimicrobia
Euglenida
Euryarchaeota
Fibrobacteres
Firmicutes
Fusobacteria
Gemmatimonadetes
Glomeromycota
Hemichordata
Korarchaeota
Lentisphaerae
Microsporidia
Mollusca
Nanoarchaeota
Nematoda
Nemertea
Nitrospirae
Phaeophyceae
Placozoa
Planctomycetes
Platyhelminthes
Porifera
Proteobacteria
Spirochaetes
Streptophyta
Synergistetes
Tenericutes
Thaumarchaeota
Thermotogae
Verrucomicrobia
Xanthophyceae
unclassified (derived from Bacteria)
unclassified (derived from Eukaryota)
unclassified (derived from Fungi)
unclassified (derived from Viruses)
unclassified (derived from other sequences)

Fig S5(a)

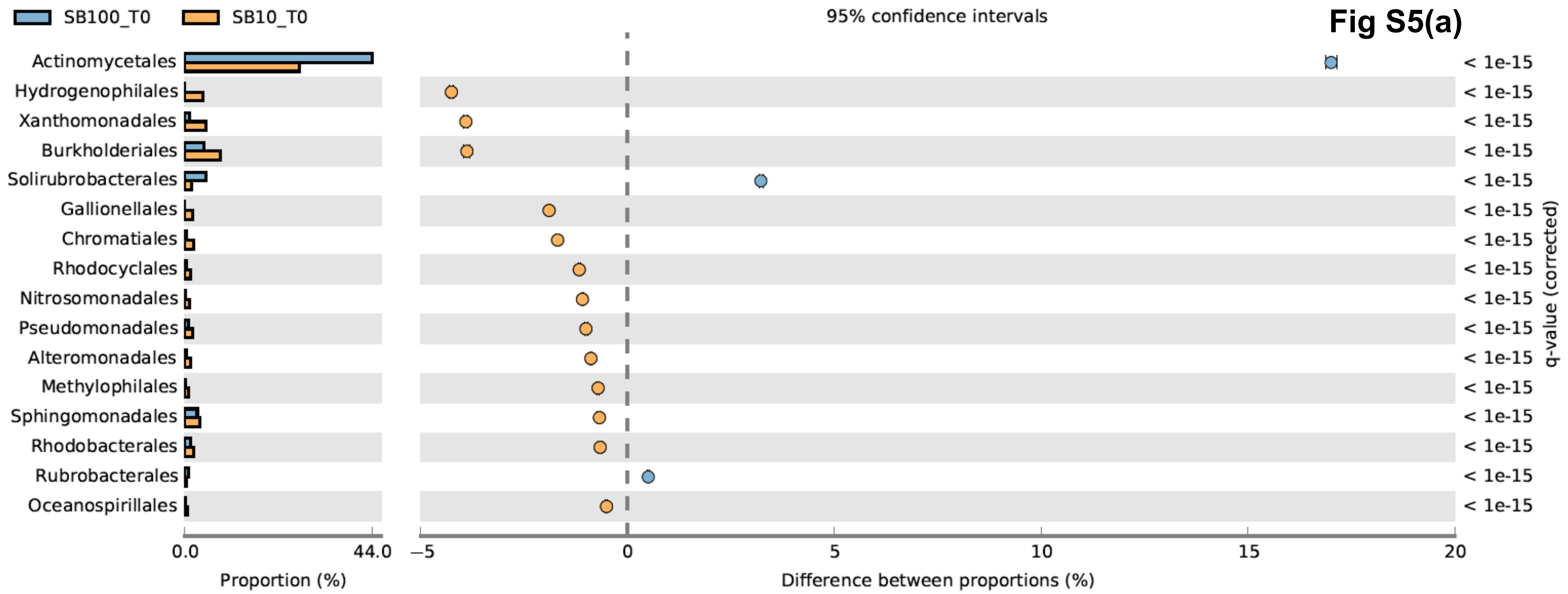

Fig S5(b)

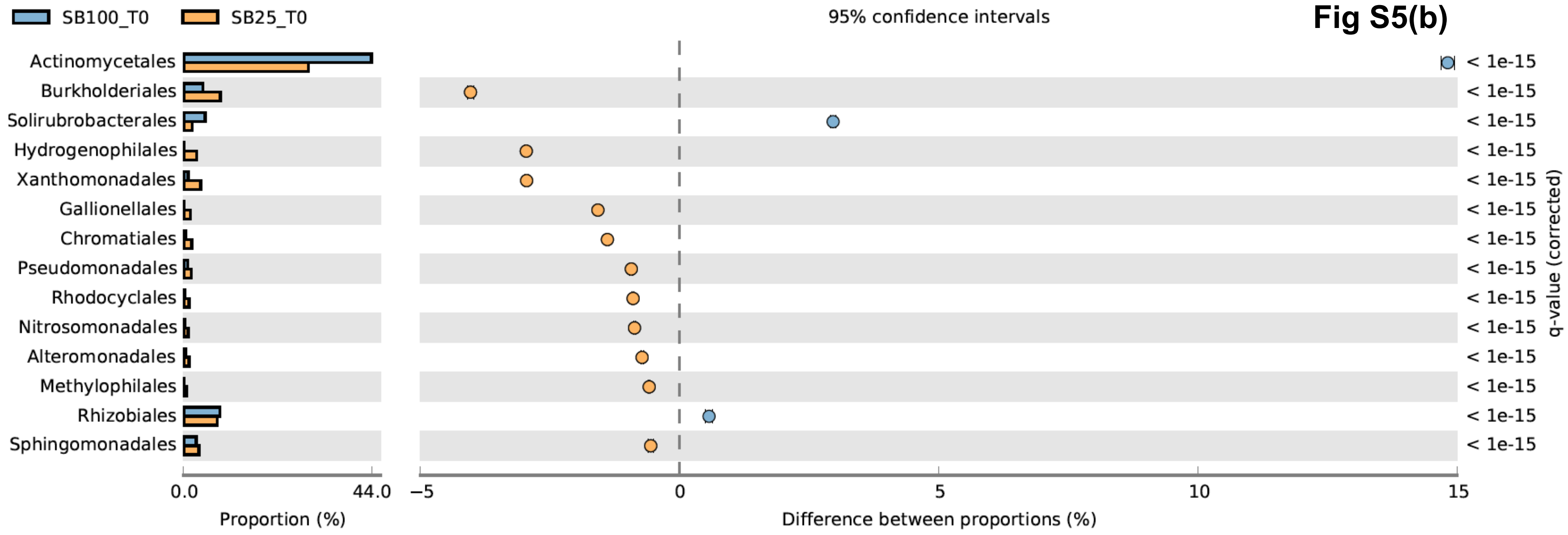

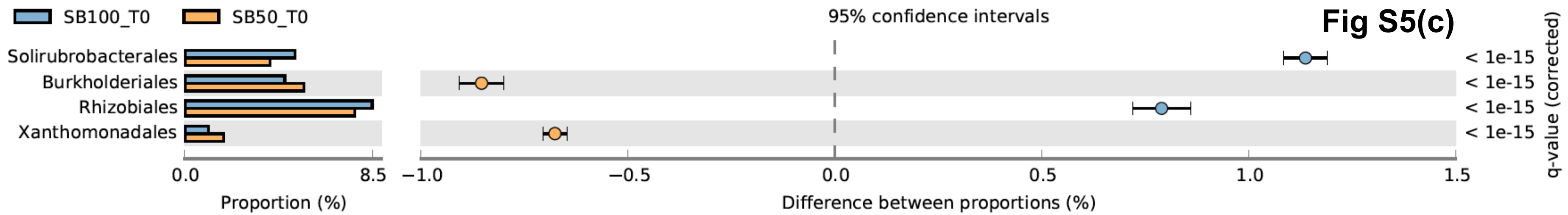
SB100_T0
SB50_T0
95% confidence intervals
Fig S5(c)
Solirubrobacterales
Burkholderiales
Rhizobiales
Xanthomonadales
< 1e-15
< 1e-15
< 1e-15
< 1e-15
q-value (corrected)
0.0
8.5
Proportion (%)
−1.0
−0.5
0.0
0.5
1.0
1.5
Difference between proportions (%)

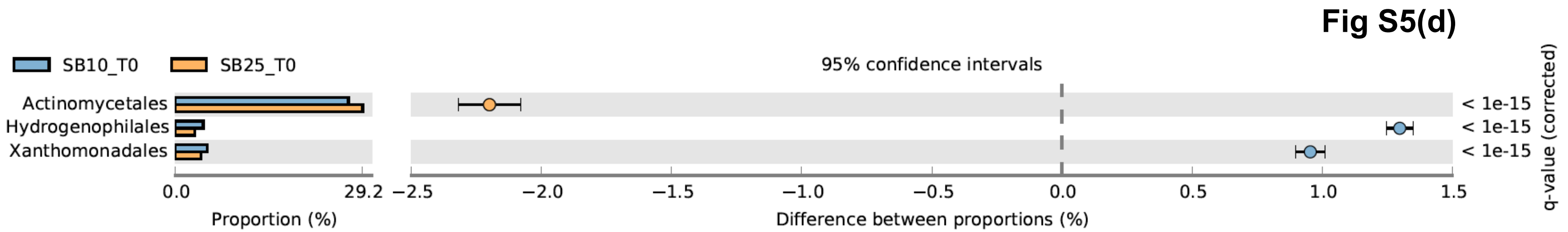
Fig S5(d)
SB10_T0
SB25_T0
95% confidence intervals
Actinomycetales
Hydrogenophilales
Xanthomonadales
< 1e-15
< 1e-15
< 1e-15
q-value (corrected)
0.0
29.2
Proportion (%)
−2.5
−2.0
−1.5
−1.0
−0.5
0.0
0.5
1.0
1.5
Difference between proportions (%)

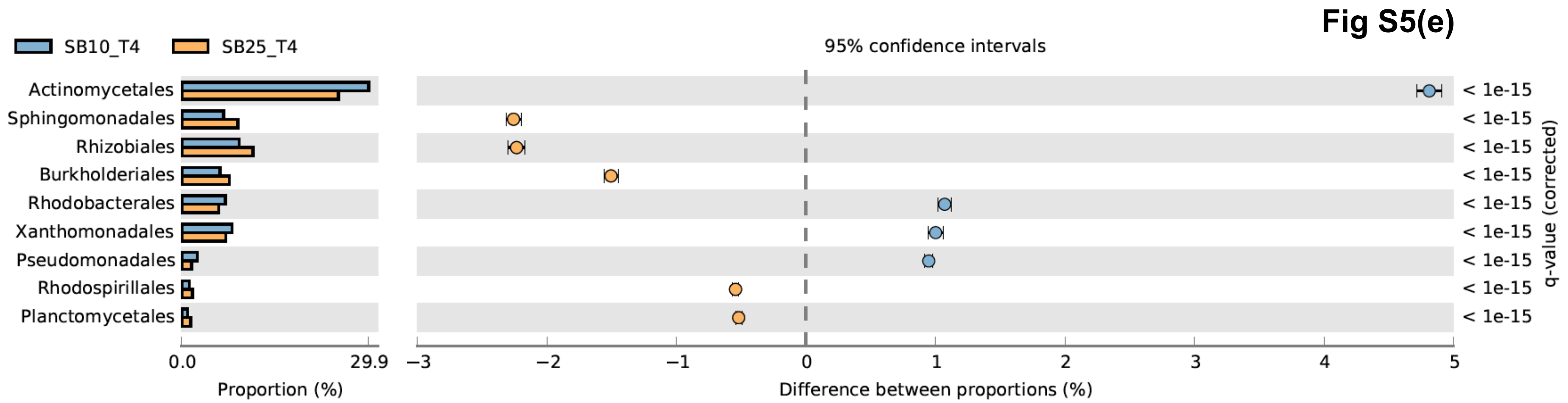
Fig S5(e)
SB10_T4
SB25_T4
95% confidence intervals
Actinomycetales
Sphingomonadales
Rhizobiales
Burkholderiales
Rhodobacterales
Xanthomonadales
Pseudomonadales
Rhodospirillales
Planctomycetales
< 1e-15
< 1e-15
< 1e-15
< 1e-15
< 1e-15
< 1e-15
< 1e-15
< 1e-15
< 1e-15
q-value (corrected)
0.0
29.9
Proportion (%)
−3
−2
−1
0
1
2
3
4
5
Difference between proportions (%)

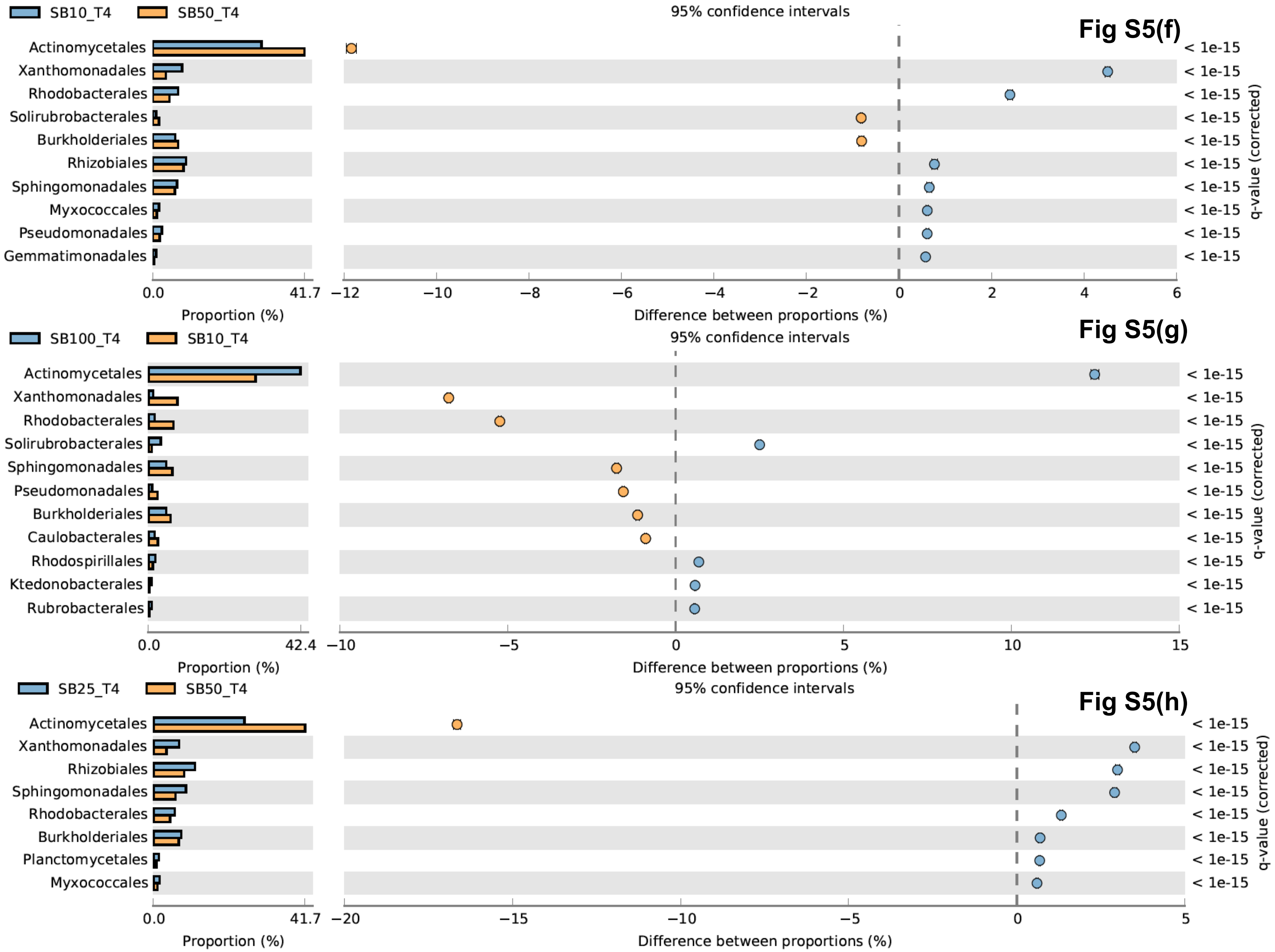
SB10_T4
SB50_T4
95% confidence intervals
Fig S5(f)
Actinomycetales
Xanthomonadales
Rhodobacterales
Solirubrobacterales
Burkholderiales
Rhizobiales
Sphingomonadales
Myxococcales
Pseudomonadales
Gemmatimonadales
0.0
41.7
Proportion (%)
−12
−10
−8
−6
−4
−2
0
2
4
6
Difference between proportions (%)
< 1e-15
q-value (corrected)
SB100_T4
SB10_T4
95% confidence intervals
Fig S5(g)
Actinomycetales
Xanthomonadales
Rhodobacterales
Solirubrobacterales
Sphingomonadales
Pseudomonadales
Burkholderiales
Caulobacterales
Rhodospirillales
Ktedonobacterales
Rubrobacterales
0.0
42.4
Proportion (%)
−10
−5
0
5
10
15
Difference between proportions (%)
< 1e-15
q-value (corrected)
SB25_T4
SB50_T4
95% confidence intervals
Fig S5(h)
Actinomycetales
Xanthomonadales
Rhizobiales
Sphingomonadales
Rhodobacterales
Burkholderiales
Planctomycetales
Myxococcales
0.0
41.7
Proportion (%)
−20
−15
−10
−5
0
5
Difference between proportions (%)
< 1e-15
q-value (corrected)

Fig S5(i)

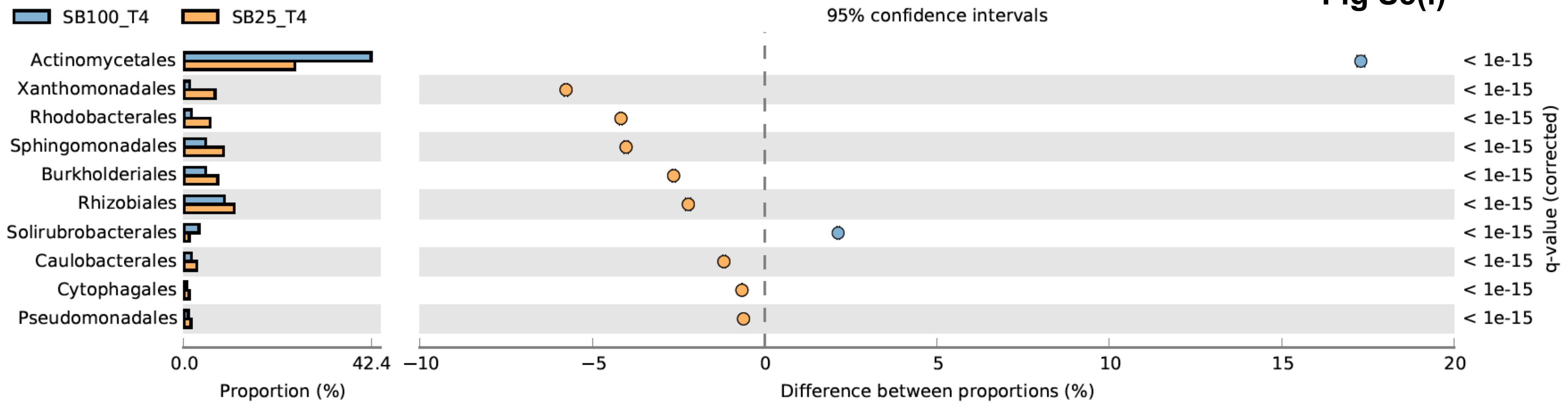

Fig S5(j)

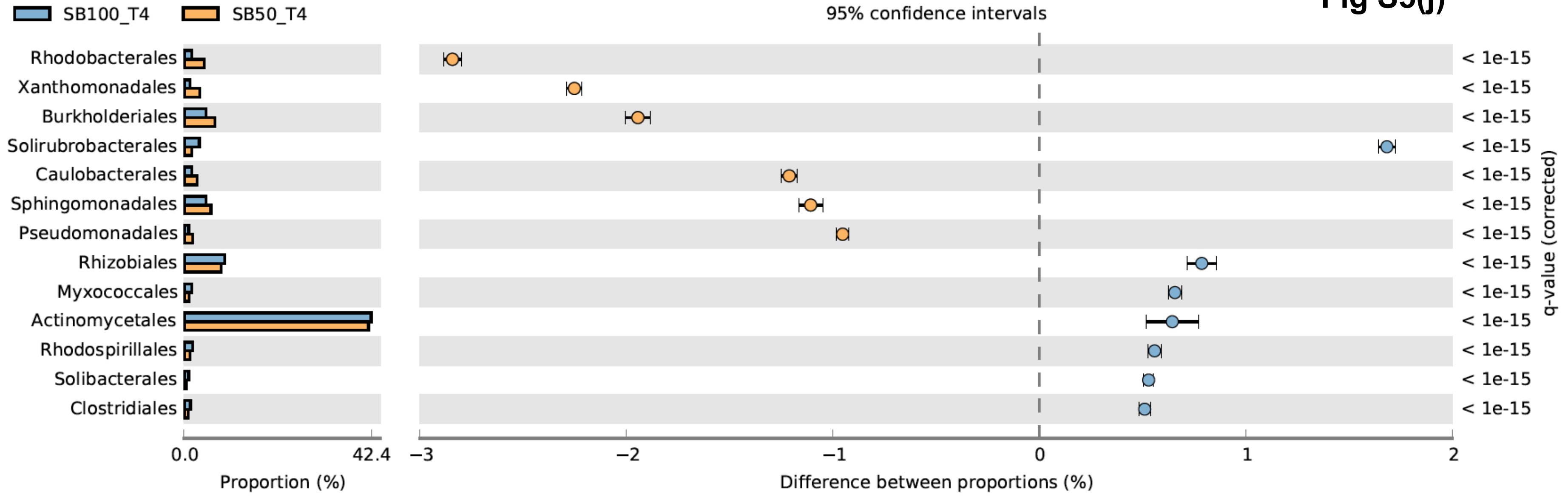

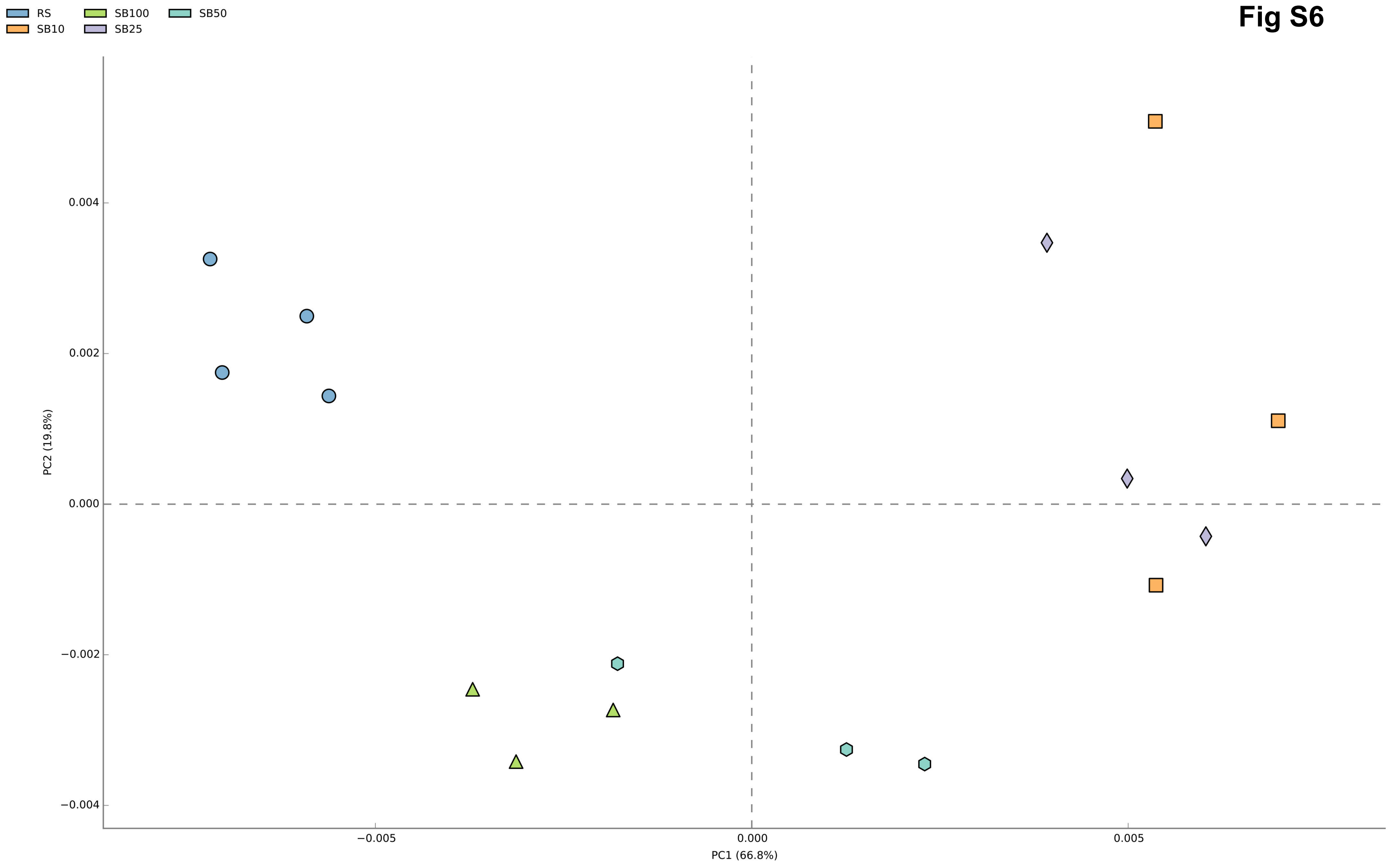
RS
SB10
SB100
SB25
SB50
0.004
0.002
0.000
−0.002
−0.004
PC2 (19.8%)
−0.005
0.000
0.005
PC1 (66.8%)

Fig S6

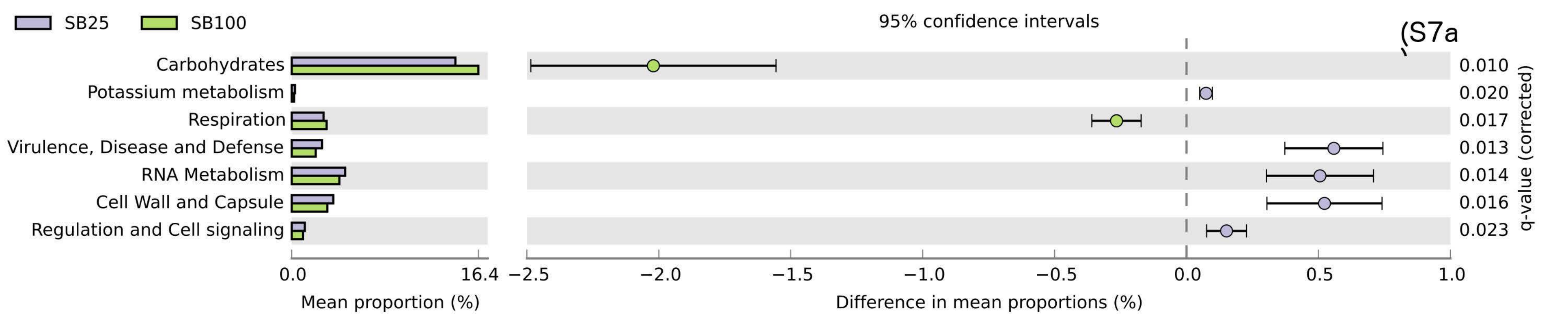

SB25
SB100
95% confidence intervals
(S7a
Carbohydrates
Potassium metabolism
Respiration
Virulence, Disease and Defense
RNA Metabolism
Cell Wall and Capsule
Regulation and Cell signaling
0.010
0.020
0.017
0.013
0.014
0.016
0.023
q-value (corrected)
0.0
16.4
Mean proportion (%)
−2.5
−2.0
−1.5
−1.0
−0.5
0.0
0.5
1.0
Difference in mean proportions (%)

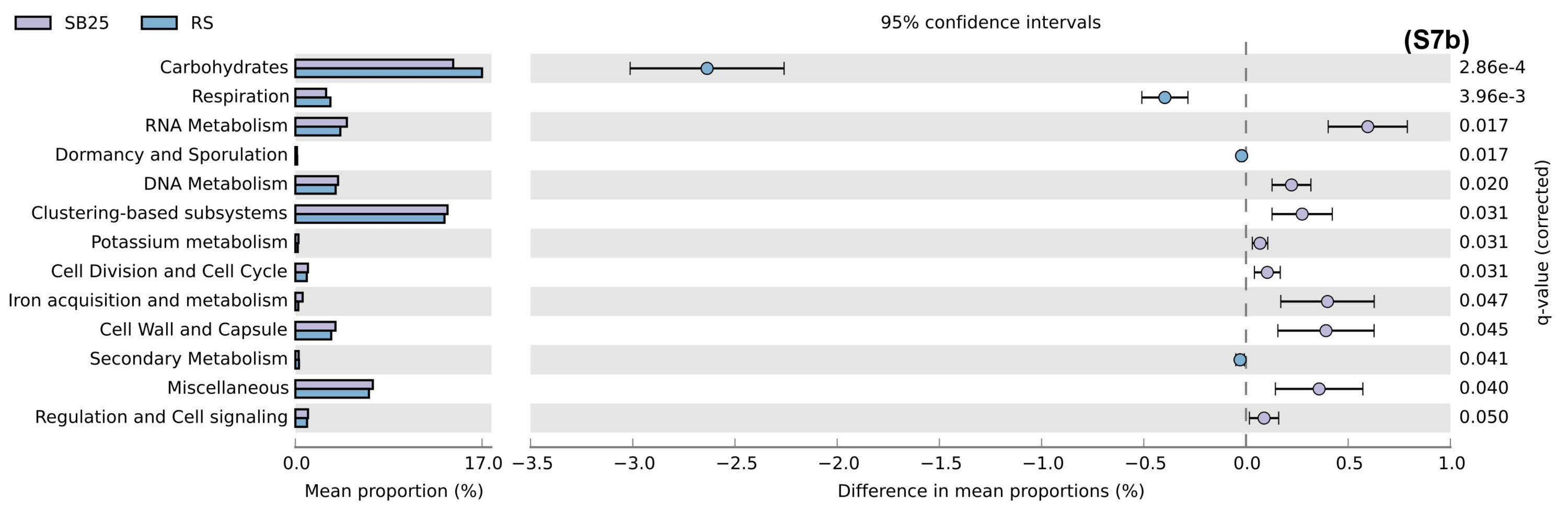
SB25
RS
95% confidence intervals
(S7b)
Carbohydrates
Respiration
RNA Metabolism
Dormancy and Sporulation
DNA Metabolism
Clustering-based subsystems
Potassium metabolism
Cell Division and Cell Cycle
Iron acquisition and metabolism
Cell Wall and Capsule
Secondary Metabolism
Miscellaneous
Regulation and Cell signaling
2.86e-4
3.96e-3
0.017
0.017
0.020
0.031
0.031
0.031
0.047
0.045
0.041
0.040
0.050
q-value (corrected)
0.0
17.0
−3.5
−3.0
−2.5
−2.0
−1.5
−1.0
−0.5
0.0
0.5
1.0
Mean proportion (%)
Difference in mean proportions (%)

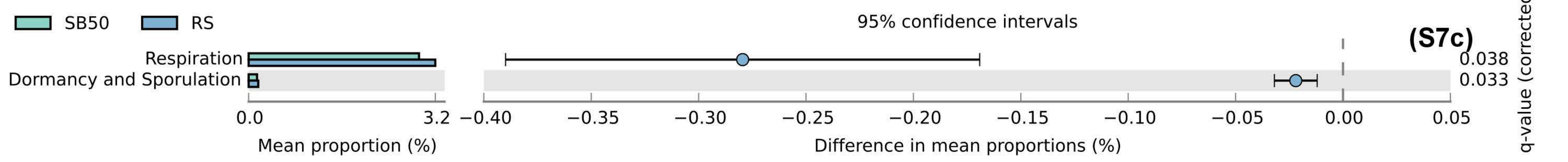
SB50
RS
95% confidence intervals
(S7c)
Respiration
Dormancy and Sporulation
0.038
0.033
q-value (corrected)
0.0
3.2
Mean proportion (%)
−0.40
−0.35
−0.30
−0.25
−0.20
−0.15
−0.10
−0.05
0.00
0.05
Difference in mean proportions (%)

	NH4-N	NO3-N	P	K	S	Org C	EC	pH	Cu	Fe	Mn	Zn	Al	Ca	Mg	Exc. K	Na	B
	mg/Kg	mg/Kg	mg/Kg	mg/Kg	mg/Kg	%	dS/m		mg/Kg	mg/Kg	mg/Kg	mg/Kg	meq/100g	meq/100g	meq/100g	meq/100g	meq/100g	mg/Kg
**T0.SB100**	11	19	4	629	119.8	0.65	0.56	5.7	1.17	13.79	20.72	0.77	0.287	2.34	0.92	1.1	1.41	1.05
**T0.SB10**	1	4	3	2785	105.4	0.33	0.37	8	1.02	47.01	11.11	1.2	0.027	3.8	0.49	1.49	0.63	0.48
**T0.SB25**	1	4	3	2668	112.7	0.41	0.4	8	1.23	44.13	12.26	1.4	0.032	4.01	0.52	1.43	0.71	0.5
**T0.SB50**	2	10	3	2189	125.5	0.46	0.48	7.8	1.07	35.8	15.04	1.05	0.059	4.39	0.6	1.42	0.91	0.65
**T1.SB100**	10	15	4	638	113.2	0.7	0.46	6.1	1.31	11.34	16.86	0.77	0.194	2.21	0.78	1.03	1.13	1.12
**T1.SB10**	1	4	3	2831	78.2	0.34	0.29	8.1	0.83	51.62	11.6	1.05	0.037	3.96	0.44	1.44	0.49	0.52
**T1.SB25**	1	6	3	2700	87.1	0.39	0.35	8	1.02	43.75	13.16	1.09	0.045	3.99	0.49	1.44	0.67	0.56
**T1.SB50**	1	8	3	2074	101.6	0.59	0.4	7.9	1.06	36.61	13.86	0.95	0.071	4.54	0.56	1.4	0.79	0.69
**T4.SB100**	5	5	3	545	40	0.46	0.15	5.9	1.11	14.76	7.55	0.52	0.186	1.86	0.56	0.87	0.5	1.46
**T4.SB10**	0	1	3	2841	35.6	0.28	0.18	8.2	1.16	50.69	10.85	1.11	0.034	3.76	0.4	1.3	0.27	0.99
**T4.SB25**	1	0	3	2578	26.8	0.33	0.18	8.2	1.04	47.99	11.63	1.02	0.051	4.04	0.41	1.31	0.3	0.87
**T4.SB50**	1	4	3	2166	29.7	0.45	0.2	8	1.05	36.85	11.47	0.78	0.081	4.22	0.46	1.28	0.38	0.85
**RS1.1**	3	0	0	90	12.3	0.61	0.016	4.3	1.48	22.39	4.31	0.72	0.897	0.42	0.13	0.21	0.03	0.48
**RS1.2**	5	1	2	82	9.1	1.24	0.021	4.4	0.94	55.7	9.1	0.57	0.655	0.83	0.29	0.21	0.05	0.65
**RS1.4**	24	16	3	93	17.8	0.51	0.039	4.4	0.73	8.41	2.49	0.45	0.622	0.34	0.11	0.22	0.08	0.39
**RS1.6**	21	33	2	72	16.4	0.61	0.076	4.2	0.85	17.51	3.72	0.46	0.696	0.62	0.15	0.19	0.17	0.54
**RS2.1**	35	5	3	137	20.5	0.98	0.8	4.3	1.08	24.51	10.39	0.91	0.545	1.04	0.79	0.31	3.16	0.69
**RS2.2**	3	0	2	84	7	0.81	0.012	4.5	1.18	21.15	7.51	0.58	0.458	1.44	0.41	0.21	0.07	0.54
**RS2.4**	4	1	2	141	8.6	1.04	0.031	4.4	1.21	16.81	15.96	0.98	0.629	0.49	0.22	0.32	0.06	0.57
**RS2.5**	3	0	0	83	14.7	0.55	0.014	4.2	0.81	13.98	1.93	0.48	1.036	0.13	0.08	0.19	0.02	0.52
**RS2.6**	2	2	2	110	7.3	0.33	0.014	4.6	0.64	9.25	6.03	0.31	0.407	0.75	0.24	0.28	0.06	0.66
**RS3.1**	6	2	3	101	11.3	1.3	0.02	4.3	0.83	46.92	4.14	0.6	0.787	0.71	0.26	0.26	0.05	0.66
**RS3.2**	10	8	3	156	13.1	1.33	0.04	4.4	1.22	29.93	16.65	0.72	0.617	0.89	0.29	0.38	0.09	0.79
**RS3.5**	3	2	3	67	14.3	0.72	0.021	4.1	0.91	22.76	1.21	0.41	1.028	0.29	0.1	0.17	0.03	0.97
**RS3.6**	2	2	2	58	17.8	0.39	0.018	4.1	0.92	12.74	1.25	0.39	1.119	0.2	0.08	0.15	0.03	0.54
**RS4.1**	39	31	5	257	27.5	0.92	0.237	4.4	1.49	26.62	6.29	1.25	0.403	1.41	0.56	0.62	0.31	1.06
**RS4.7**	3	1	3	150	10.4	1.01	0.034	4.5	1.4	9.3	20.22	0.68	0.57	0.58	0.3	0.35	0.13	0.69

Chemical Characteristics of topsoil and tailings used for substrate blend prepration

	EC	pH	OrgC	N	P	Al	B	Ca	Cu	Fe	K	Mg	Mn	Na	P	S	Zn
**Units**	mS/m		%	%	mg/kg	mg/kg	mg/kg	mg/kg	mg/kg	mg/kg	mg/kg	mg/kg	mg/kg	mg/kg	mg/kg	mg/kg	mg/kg
**Topsoil (stored)**	18	6.7	0.69	0.028	240	>550	1	470	2.2	58	370	83	52	120	16	61	1
**Tailings**	16	9.7	0.2	<0.005	1100	59	0.4	4000	1.5	550	550	150	48	46	6	21	3.5

Factor	Treatment			
	**SB10**	**SB25**	**SB50**	**SB100**
Ammonium Nitrogen	0.83b	1.00b	1.33b	7.50a
Nitrate Nitrogen	3b	5ab	7.33ab	10a
Phosphorus (Colwell)	3a	3a	3a	3.33a
Potassium (Colwell)	2819a	2648.67b	2143c	591.33d
Sulphur	73.07a	75.53a	85.6a	71.4a
Organic Carbon	0.31c	0.37bc	0.5ab	0.61a
Conductivity	0.28a	0.31a	0.36a	0.39a
pH ($CaCl_2$)	8.1a	8.06a	7.9a	6.23b
Copper	1a	1.09a	1.06a	1.54a
Iron	49.77a	45.29a	36.42a	28.03a
Manganese	11.18a	12.35a	13.45a	25.47a
Zinc	1.12a	1.17a	0.92ab	0.76b
Exc. Aluminium	0.03b	0.04b	0.07b	0.31a
Exc. Calcium	3.84b	4.01b	4.38a	2.03c
Exc. Magnesium	0.44b	0.47b	0.54b	0.72a
Exc. Potassium	1.41a	1.39a	1.36a	0.95b
Exc. Sodium	0.46a	0.56a	0.69a	0.81a
Boron	0.66b	0.64b	0.73b	1.19a

Table S3 Similarity percentage (SIMPER) analysis of major taxa (at the level of Order) contributing to dissimilarity between different substrate blends.

Groups SB100 & SB10						
Average dissimilarity = 33.85						
	Group 100	Group 10				
Taxa	Av.Abund	Av.Abund	Av.Diss	Diss/SD	Contrib%	Cum.%
Xanthomonadales	0.15	0.38	2.34	2	6.92	6.92
Rhodocyclales	0.2	0.06	2.02	1.03	5.98	12.9
Sphingobacteriales	0.27	0.39	1.39	1.46	4.09	16.99
Actinomycetales	0.4	0.39	1.25	1.45	3.69	20.69
Rhodobacterales	0.03	0.15	1.22	1.64	3.62	24.31
Bacteria;Other	0.36	0.24	1.21	1.6	3.58	27.88
Thermoprotei;Other	0.14	0.05	1.15	1.09	3.4	31.28
Burkholderiales	0.22	0.31	0.98	1.52	2.89	34.18
Ohtaekwangia	0.03	0.13	0.95	1.24	2.81	36.99
Sphingomonadales	0.24	0.21	0.88	1.17	2.6	39.59
BetaproteoOther	0.15	0.11	0.79	1.23	2.35	41.94
Acidobacteria_Gp3;Gp3	0.13	0.08	0.78	1.34	2.29	44.23
Solirubrobacterales	0.18	0.13	0.73	1.66	2.17	46.4
Caulobacterales	0.08	0.14	0.69	1.91	2.04	48.44
Rhodospirillales	0.15	0.11	0.64	1.34	1.89	50.33
Hydrogenophilales	0.06	0.06	0.62	1.22	1.83	52.15
Bacillales	0.11	0.09	0.58	1.54	1.73	53.88
Acidobacteria_Gp6;Gp6	0.03	0.09	0.58	1.63	1.72	55.6
GammaproteoOther	0.07	0.09	0.55	1.96	1.63	57.23
KtedonoOther	0.08	0.06	0.54	1.35	1.6	58.83
Acidobacteria_Gp3;Other	0.07	0.03	0.53	1.45	1.55	60.38
ActinoOther	0.15	0.12	0.49	1.92	1.45	61.83
Verrucomicrobia;Subdivision3;Subdivision3_genera_incertae_sedis	0.06	0.09	0.47	1.43	1.39	63.22
Pseudomonadales	0.06	0.09	0.45	1.42	1.34	64.56
Acidobacteria_Gp1;Gp1	0.06	0.02	0.45	1.84	1.33	65.89
Acidobacteria_Gp4;Gp4	0.05	0.02	0.45	1.14	1.32	67.21
Acidobacteria_Gp2;Gp2	0.04	0	0.43	0.4	1.26	68.47
Ktedonobacterales	0.04	0.02	0.4	1.22	1.19	69.67

Flavobacteriales	0.05	0.02	0.4	1.19	1.19	70.86
Groups SB100 & SB25						
Average dissimilarity = 32.72						
	Group 100	Group 25				
Taxa	Av.Abund	Av.Abund	Av.Diss	Diss/SD	Contrib%	Cum.%
Rhodocyclales	0.2	0.08	2.08	1.05	6.36	6.36
Xanthomonadales	0.15	0.32	1.7	1.98	5.18	11.55
Sphingobacteriales	0.27	0.36	1.33	1.2	4.08	15.62
Actinomycetales	0.4	0.44	1.25	1.36	3.82	19.44
Thermoprotei;Other	0.14	0.05	1.11	1.09	3.4	22.84
Hydrogenophilales	0.06	0.1	1.1	0.93	3.36	26.21
Bacteria;Other	0.36	0.27	1.01	1.43	3.08	29.28
Burkholderiales	0.22	0.31	0.97	1.4	2.97	32.26
BetaproteoOther	0.15	0.09	0.93	1.17	2.85	35.11
Sphingomonadales	0.24	0.22	0.91	1.2	2.77	37.88
Pseudomonadales	0.06	0.12	0.79	1.31	2.42	40.3
Acidobacteria_Gp3;Gp3	0.13	0.09	0.76	1.67	2.33	42.62
GammaproteoOther	0.07	0.1	0.74	1.05	2.25	44.87
Solirubrobacterales	0.18	0.14	0.71	1.47	2.16	47.04
Rhodospirillales	0.15	0.14	0.62	1.52	1.9	48.94
Ohtaekwangia	0.03	0.07	0.59	1.21	1.81	50.75
Rhodobacterales	0.03	0.08	0.58	1.58	1.77	52.52
Acidobacteria_Gp3;Other	0.07	0.02	0.57	1.49	1.74	54.25
KtedonoOther	0.08	0.06	0.56	1.71	1.71	55.96
Bacillales	0.11	0.11	0.5	1.34	1.53	57.49
Gemmatimonadales	0.09	0.08	0.47	1.38	1.45	58.93
ActinoOther	0.15	0.14	0.44	1.58	1.35	60.28
Acidobacteria_Gp2;Gp2	0.04	0	0.43	0.4	1.32	61.6
Acidobacteria_Gp4;Gp4	0.05	0.02	0.43	1.43	1.31	62.91
Acidobacteria_Gp6;Gp6	0.03	0.07	0.42	1.4	1.28	64.19
Flavobacteriales	0.05	0.03	0.41	1.21	1.26	65.46
Ktedonobacterales	0.04	0.02	0.4	1.26	1.23	66.69
Bdellovibrionales	0.04	0.05	0.39	1.39	1.2	67.89
Acidobacteria_Gp1;Gp1	0.06	0.04	0.38	1.64	1.17	69.06

Alteromonadales	0.02	0.04	0.38	1.23	1.17	70.23
Groups SB10 & SB25						
Average dissimilarity = 25.38						
	Group 10	Group 25				
Taxa	Av.Abund	Av.Abund	Av.Diss	Diss/SD	Contrib%	Cum.%
Sphingobacteriales	0.39	0.36	1.28	1.51	5.05	5.05
Actinomycetales	0.39	0.44	1.08	1.34	4.25	9.3
Hydrogenophilales	0.06	0.1	1.06	0.85	4.16	13.47
Xanthomonadales	0.38	0.32	1.03	1.2	4.05	17.52
Ohtaekwangia	0.13	0.07	0.92	1.29	3.63	21.15
Rhodobacterales	0.15	0.08	0.83	1.27	3.27	24.42
Solirubrobacterales	0.13	0.14	0.81	1.4	3.18	27.6
Rhodocyclales	0.06	0.08	0.8	1.03	3.13	30.73
Pseudomonadales	0.09	0.12	0.69	1.42	2.71	33.44
Burkholderiales	0.31	0.31	0.67	1.47	2.64	36.07
GammaproteoOther	0.09	0.1	0.65	1.14	2.56	38.64
Bacteria;Other	0.24	0.27	0.58	1.28	2.29	40.93
Verrucomicrobia;Subdivision3;Subdivision3_genera_incertae_sedis	0.09	0.05	0.57	1.43	2.24	43.16
ActinoOther	0.12	0.14	0.54	1.36	2.13	45.3
Bacillales	0.09	0.11	0.54	1.53	2.11	47.41
Sphingomonadales	0.21	0.22	0.51	1.26	1.99	49.4
BetaproteoOther	0.11	0.09	0.48	2.55	1.88	51.28
Gemmatimonadales	0.09	0.08	0.48	1.4	1.87	53.16
Bdellovibrionales	0.06	0.05	0.47	1.35	1.85	55.01
Rhodospirillales	0.11	0.14	0.47	1.33	1.84	56.86
KtedonoOther	0.06	0.06	0.44	1.38	1.75	58.61
Caulobacterales	0.14	0.11	0.42	1.38	1.64	60.24
Acidobacteria_Gp7;Gp7	0.05	0.01	0.41	1.75	1.61	61.85
Acidobacteria_Gp3;Gp3	0.08	0.09	0.38	1.44	1.5	63.35
Thermoprotei;Other	0.05	0.05	0.35	1.46	1.37	64.72
Acidobacteria_Gp6;Gp6	0.09	0.07	0.34	1.31	1.34	66.06
Alteromonadales	0.04	0.04	0.34	1.33	1.34	67.41
Bacteroidetes;Other;Other	0.06	0.04	0.34	1.16	1.34	68.75

ProteoOther;Other	0.09	0.1	0.33	1.41	1.3	70.04
Groups SB100 & SB50						
Average dissimilarity = 29.85						
	Group 100	Group 50				
Taxa	Av.Abund	Av.Abund	Av.Diss	Diss/SD	Contrib%	Cum.%
Rhodocyclales	0.2	0.04	1.96	0.93	6.58	6.58
Sphingobacteriales	0.27	0.45	1.84	1.55	6.16	12.74
Xanthomonadales	0.15	0.26	1.27	1.71	4.24	16.98
Actinomycetales	0.4	0.41	1.16	1.43	3.87	20.86
Burkholderiales	0.22	0.32	0.98	1.73	3.28	24.13
Thermoprotei;Other	0.14	0.08	0.95	1.04	3.2	27.33
BetaproteoOther	0.15	0.08	0.89	1.11	2.98	30.32
Sphingomonadales	0.24	0.19	0.86	1.06	2.89	33.21
Bacteria;Other	0.36	0.29	0.83	1.63	2.77	35.98
Pseudomonadales	0.06	0.12	0.71	1.04	2.39	38.37
Acidobacteria_Gp3;Gp3	0.13	0.14	0.66	1.7	2.22	40.59
Rhodospirillales	0.15	0.17	0.57	1.69	1.91	42.5
Hydrogenophilales	0.06	0.02	0.56	1.09	1.88	44.39
KtedonoOther	0.08	0.1	0.53	1.83	1.79	46.18
Bacteroidetes;Other;Other	0.04	0.09	0.52	1.7	1.74	47.92
Solirubrobacterales	0.18	0.14	0.51	1.45	1.7	49.62
Rhizobiales	0.17	0.2	0.48	2.15	1.61	51.23
Bdellovibrionales	0.04	0.08	0.48	1.26	1.6	52.82
Bacillales	0.11	0.11	0.48	1.39	1.59	54.41
Rhodobacterales	0.03	0.07	0.46	1.29	1.56	55.97
GammaproteoOther	0.07	0.06	0.43	1.22	1.44	57.41
Flavobacteriales	0.05	0.07	0.43	1.65	1.43	58.84
Acidobacteria_Gp2;Gp2	0.04	0	0.42	0.4	1.41	60.25
Acidobacteria_Gp4;Gp4	0.05	0.03	0.41	1.68	1.39	61.64
Ktedonobacterales	0.04	0.04	0.41	1.7	1.38	63.01
Acidobacteria_Gp3;Other	0.07	0.06	0.41	1.91	1.36	64.37
Chloroplast	0.02	0.04	0.39	1.17	1.32	65.69
Caulobacterales	0.08	0.11	0.39	1.38	1.31	67
ActinoOther	0.15	0.11	0.39	1.76	1.29	68.29

Gemmatimonadales	0.09	0.11	0.37	1.41	1.24	69.54
ProteoOther;Other	0.1	0.07	0.37	1.34	1.24	70.78
Groups SB10 & SB50						
Average dissimilarity = 24.56						
	Group 10	Group 50				
Taxa	Av.Abund	Av.Abund	Av.Diss	Diss/SD	Contrib%	Cum.%
Xanthomonadales	0.38	0.26	1.4	1.43	5.69	5.69
Sphingobacteriales	0.39	0.45	1.1	1.27	4.48	10.17
Rhodobacterales	0.15	0.07	0.92	1.36	3.75	13.92
Actinomycetales	0.39	0.41	0.9	1.3	3.67	17.59
Ohtaekwangia	0.13	0.06	0.77	1.17	3.12	20.71
Acidobacteria_Gp3;Gp3	0.08	0.14	0.69	1.71	2.82	23.54
Solirubrobacterales	0.13	0.14	0.63	1.59	2.58	26.11
Pseudomonadales	0.09	0.12	0.63	1.13	2.57	28.68
Bacteria;Other	0.24	0.29	0.63	1.84	2.56	31.24
Rhodospirillales	0.11	0.17	0.6	1.83	2.44	33.68
Burkholderiales	0.31	0.32	0.52	1.45	2.13	35.81
Bacillales	0.09	0.11	0.51	1.37	2.06	37.87
Thermoprotei;Other	0.05	0.08	0.5	1.55	2.02	39.89
Hydrogenophilales	0.06	0.02	0.5	1.01	2.02	41.91
KtedonoOther	0.06	0.1	0.5	1.37	2.02	43.93
Bdellovibrionales	0.06	0.08	0.49	1.32	1.99	45.92
Caulobacterales	0.14	0.11	0.49	1.43	1.98	47.9
Flavobacteriales	0.02	0.07	0.47	1.75	1.92	49.82
Rhodocyclales	0.06	0.04	0.46	1.07	1.88	51.7
Verrucomicrobia;Subdivision3;Subdivision3_genera_incertae_sedis	0.09	0.07	0.45	1.56	1.82	53.52
Acidobacteria_Gp6;Gp6	0.09	0.05	0.44	1.56	1.79	55.31
Rhizobiales	0.18	0.2	0.44	2.09	1.78	57.09
Chloroplast	0	0.04	0.41	1.05	1.68	58.77
Sphingomonadales	0.21	0.19	0.4	1.17	1.65	60.41
ActinoOther	0.12	0.11	0.39	1.33	1.58	61.99
GammaproteoOther	0.09	0.06	0.38	1.53	1.55	63.55
Acidobacteria_Gp3;Other	0.03	0.06	0.38	1.49	1.55	65.1

ProteoOther;Other	0.09	0.07	0.36	1.37	1.47	66.57
Bacteroidetes;Other;Other	0.06	0.09	0.36	1.39	1.46	68.03
Gemmatimonadales	0.09	0.11	0.35	1.4	1.44	69.46
Acidobacteria_Gp4;Gp4	0.02	0.03	0.35	1.6	1.42	70.89
Groups SB25 & SB50						
Average dissimilarity = 24.24						
	Group 25	Group 50				
Taxa	Av.Abund	Av.Abund	Av.Diss	Diss/SD	Contrib%	Cum.%
Sphingobacteriales	0.36	0.45	1.47	1.45	6.07	6.07
Hydrogenophilales	0.1	0.02	0.95	0.7	3.92	9.99
Actinomycetales	0.44	0.41	0.91	1.38	3.77	13.76
Xanthomonadales	0.32	0.26	0.82	1.2	3.39	17.15
Pseudomonadales	0.12	0.12	0.82	1.34	3.37	20.52
Rhodocyclales	0.08	0.04	0.71	0.86	2.93	23.46
Solirubrobacterales	0.14	0.14	0.67	1.52	2.77	26.23
Burkholderiales	0.31	0.32	0.61	1.54	2.52	28.75
Ohtaekwangia	0.07	0.06	0.59	1.68	2.42	31.17
GammaproteoOther	0.1	0.06	0.58	0.79	2.38	33.55
Acidobacteria_Gp3;Gp3	0.09	0.14	0.57	1.46	2.36	35.9
Bacteroidetes;Other;Other	0.04	0.09	0.51	1.48	2.11	38.01
Gemmatimonadales	0.08	0.11	0.49	1.28	2.04	40.05
Bacteria;Other	0.27	0.29	0.49	2.05	2.02	42.07
Bdellovibrionales	0.05	0.08	0.48	1.27	1.96	44.03
Sphingomonadales	0.22	0.19	0.47	1.14	1.96	45.99
Rhodospirillales	0.14	0.17	0.47	1.47	1.95	47.93
ActinoOther	0.14	0.11	0.45	1.42	1.87	49.8
Rhizobiales	0.18	0.2	0.45	1.38	1.86	51.65
Flavobacteriales	0.03	0.07	0.44	1.47	1.82	53.47
KtedonoOther	0.06	0.1	0.43	1.12	1.79	55.26
Bacillales	0.11	0.11	0.43	1.39	1.78	57.04
Chloroplast	0	0.04	0.43	1.04	1.78	58.82
Acidobacteria_Gp3;Other	0.02	0.06	0.43	1.57	1.77	60.58
ProteoOther;Other	0.1	0.07	0.39	1.52	1.61	62.19
Rhodobacterales	0.08	0.07	0.39	1.32	1.6	63.8

Thermoprotei;Other	0.05	0.08	0.38	1.45	1.56	65.36
Verrucomicrobia;Subdivision3;Subdivision3_genera_incertae_sedis	0.05	0.07	0.36	1.39	1.5	66.85
Alteromonadales	0.04	0.03	0.35	1.29	1.45	68.3
Caulobacterales	0.11	0.11	0.33	2.01	1.36	69.66
BetaproteoOther	0.09	0.08	0.31	0.87	1.3	70.96
Groups SB100 & R						
Average dissimilarity = 45.58						
	Group 100	Group R				
Taxa	Av.Abund	Av.Abund	Av.Diss	Diss/SD	Contrib%	Cum.%
Bacteria;Other	0.36	0.67	3.4	3.72	7.46	7.46
Rhodocyclales	0.2	0.1	2.22	1.19	4.88	12.34
Actinomycetales	0.4	0.2	2.22	1.63	4.88	17.21
Sphingomonadales	0.24	0.06	1.96	1.61	4.29	21.51
Burkholderiales	0.22	0.04	1.95	3.29	4.28	25.79
Sphingobacteriales	0.27	0.1	1.84	2.04	4.03	29.82
Xanthomonadales	0.15	0	1.61	2.37	3.53	33.36
Thermoprotei;Other	0.14	0.21	1.33	1.54	2.91	36.27
Solirubrobacterales	0.18	0.27	1.29	1.29	2.84	39.11
Ktedonobacterales	0.04	0.16	1.28	2.04	2.81	41.91
Chloroflexi;Other;Other	0.04	0.14	1.12	2.46	2.45	44.37
BetaproteoOther	0.15	0.07	1.07	1.2	2.34	46.71
KtedonoOther	0.08	0.17	1.05	1.54	2.29	49
Acidobacteria_Gp3;Gp3	0.13	0.19	1.02	1.47	2.25	51.25
ActinoOther	0.15	0.23	0.96	1.38	2.1	53.35
Bacillales	0.11	0.05	0.87	1.65	1.91	55.26
Acidobacteria_Gp3;Other	0.07	0.11	0.79	1.38	1.74	56.99
partobacteria_genera_incertae_sedis	0.04	0.1	0.75	1.14	1.65	58.64
Unclassified	0.02	0.08	0.71	1.38	1.56	60.2
Rhodospirillales	0.15	0.11	0.7	1.44	1.54	61.74
Caulobacterales	0.08	0.01	0.67	2.34	1.47	63.22
Hydrogenophilales	0.06	0	0.66	1.05	1.46	64.68
Gemmatimonadales	0.09	0.03	0.66	1.69	1.45	66.12

GammaproteoOther	0.07	0.01	0.63	1.2	1.38	67.5
Verrucomicrobia;Subdivision3;Subdivision3_genera_incertae_sedis	0.06	0.01	0.61	1.93	1.34	68.84
Acidobacteria_Gp2;Gp2	0.04	0.02	0.59	0.57	1.3	70.14
Groups SB10 & R						
Average dissimilarity = 56.23						
	Group 10	Group R				
Taxa	Av.Abund	Av.Abund	Av.Diss	Diss/SD	Contrib%	Cum.%
Bacteria;Other	0.24	0.67	4.7	5.82	8.35	8.35
Xanthomonadales	0.38	0	4.17	3.58	7.42	15.77
Sphingobacteriales	0.39	0.1	3.21	2.71	5.7	21.47
Burkholderiales	0.31	0.04	3.03	3.77	5.39	26.86
Actinomycetales	0.39	0.2	2.07	2.07	3.67	30.53
Thermoprotei;Other	0.05	0.21	1.75	2.26	3.11	33.65
Solirubrobacterales	0.13	0.27	1.71	1.39	3.04	36.69
Rhodobacterales	0.15	0	1.61	2.07	2.86	39.55
Sphingomonadales	0.21	0.06	1.57	2.35	2.79	42.35
Ktedonobacterales	0.02	0.16	1.49	2.76	2.64	44.99
Caulobacterales	0.14	0.01	1.42	3.1	2.53	47.52
Ohtaekwangia	0.13	0	1.37	1.68	2.44	49.96
Acidobacteria_Gp3;Gp3	0.08	0.19	1.31	1.67	2.32	52.29
ActinoOther	0.12	0.23	1.26	1.53	2.23	54.52
Chloroflexi;Other;Other	0.03	0.14	1.23	2.92	2.2	56.71
KtedonoOther	0.06	0.17	1.22	1.87	2.18	58.89
Acidobacteria_Gp3;Other	0.03	0.11	1.01	1.5	1.8	60.69
Pseudomonadales	0.09	0.01	0.93	2.5	1.65	62.34
Verrucomicrobia;Subdivision3;Subdivision3_genera_incertae_sedis	0.09	0.01	0.92	1.97	1.64	63.98
Acidobacteria_Gp6;Gp6	0.09	0.01	0.89	2.79	1.58	65.56
GammaproteoOther	0.09	0.01	0.87	2.95	1.54	67.11
Unclassified	0	0.08	0.86	1.69	1.53	68.63
Rhodocyclales	0.06	0.1	0.84	1.08	1.5	70.13

Groups SB25 & R						
Average dissimilarity = 53.55						
	Group 25	Group R				
Taxa	Av.Abund	Av.Abund	Av.Diss	Diss/SD	Contrib%	Cum.%
Bacteria;Other	0.27	0.67	4.48	5.71	8.36	8.36
Xanthomonadales	0.32	0	3.5	5.43	6.53	14.89
Burkholderiales	0.31	0.04	3.01	3.23	5.62	20.51
Sphingobacteriales	0.36	0.1	2.94	1.88	5.48	25.99
Actinomycetales	0.44	0.2	2.72	2.55	5.07	31.06
Thermoprotei;Other	0.05	0.21	1.7	2.42	3.17	34.23
Sphingomonadales	0.22	0.06	1.69	2.27	3.15	37.38
Solirubrobacterales	0.14	0.27	1.67	1.35	3.11	40.49
Ktedonobacterales	0.02	0.16	1.52	2.97	2.83	43.32
Chloroflexi;Other;Other	0.03	0.14	1.26	2.49	2.34	45.67
Pseudomonadales	0.12	0.01	1.25	1.61	2.34	48.01
KtedonoOther	0.06	0.17	1.23	1.98	2.29	50.29
Acidobacteria_Gp3;Gp3	0.09	0.19	1.21	1.58	2.25	52.55
ActinoOther	0.14	0.23	1.15	1.41	2.15	54.69
Acidobacteria_Gp3;Other	0.02	0.11	1.07	1.52	2	56.7
Caulobacterales	0.11	0.01	1.04	3.32	1.95	58.65
Hydrogenophilales	0.1	0	1.03	0.65	1.92	60.56
Rhodocyclales	0.08	0.1	1.02	1.07	1.91	62.47
partobacteria_genera_incertae_sedis	0.02	0.1	0.93	1.22	1.74	64.21
GammaproteoOther	0.1	0.01	0.92	1.03	1.71	65.92
Rhodobacterales	0.08	0	0.88	2.63	1.65	67.57
Bacillales	0.11	0.05	0.84	1.69	1.56	69.13
Unclassified	0.01	0.08	0.83	1.58	1.56	70.69

Groups SB50 & R						
Average dissimilarity = 50.10						
	Group 50	Group R				
Taxa	Av.Abund	Av.Abund	Av.Diss	Diss/SD	Contrib%	Cum.%
Bacteria;Other	0.29	0.67	4.13	8.22	8.25	8.25
Sphingobacteriales	0.45	0.1	3.82	3.03	7.62	15.87
Burkholderiales	0.32	0.04	3.02	4.48	6.02	21.89
Xanthomonadales	0.26	0	2.83	3.63	5.65	27.54
Actinomycetales	0.41	0.2	2.28	2.61	4.55	32.09
Solirubrobacterales	0.14	0.27	1.55	1.31	3.09	35.18
Sphingomonadales	0.19	0.06	1.37	2.4	2.74	37.92
Thermoprotei;Other	0.08	0.21	1.33	1.93	2.65	40.57
ActinoOther	0.11	0.23	1.27	1.61	2.54	43.12
Ktedonobacterales	0.04	0.16	1.24	2.49	2.48	45.6
Pseudomonadales	0.12	0.01	1.21	1.53	2.43	48.03
Chloroflexi;Other;Other	0.03	0.14	1.16	2.97	2.31	50.34
Caulobacterales	0.11	0.01	1	2.32	2	52.34
Gemmatimonadales	0.11	0.03	0.92	2.84	1.84	54.18
KtedonoOther	0.1	0.17	0.87	1.63	1.73	55.91
Bdellovibrionales	0.08	0	0.86	2.09	1.71	57.63
Bacillales	0.11	0.05	0.85	1.7	1.69	59.32
Acidobacteria_Gp3;Gp3	0.14	0.19	0.85	1.41	1.69	61.01
Bacteroidetes;Other;Other	0.09	0.01	0.84	2.68	1.67	62.68
Rhodocyclales	0.04	0.1	0.83	0.99	1.66	64.34
Rhizobiales	0.2	0.14	0.79	1.7	1.58	65.93
Unclassified	0.01	0.08	0.76	1.47	1.51	67.44
Acidobacteria_Gp3;Other	0.06	0.11	0.75	1.37	1.49	68.93
partobacteria_genera_incertae_sedis	0.04	0.1	0.73	1.08	1.45	70.38
